# Extract of camellia seed cake ameliorates glycolipid metabolism disorder in mice through inhibiting ACOX1 activity^☆^

**DOI:** 10.1016/j.fochx.2025.102707

**Published:** 2025-06-30

**Authors:** Bolin Chen, Li Ma, Xinzhi Chen, Zhigang Li, Qinhe Zhu, Changwei Liu, Haixiang He, Zhixu Zhang, Chuyi Zhou, Guanying Liu, Yuqiao Zhou, Senwen Deng, Shiyin Guo, Yongzhong Chen

**Affiliations:** a Hunan Engineering Research Center of Lotus Deep Processing and Nutritional Health Sciences, School of Life and Health Sciences, Hunan University of Science and Technology, Xiangtan 411201, China; b National Engineering Research Center of Oiltea Camellia, State Key Laboratory of Utilization of Woody Oil Resources, Hunan Academy of Forestry, Shao Shan South Road, No. 658, Changsha 410004, China; c Hunan Xianglian Engineering Technology Research Center, Xiangtan 411201, China; d College of Horticulture, Hunan Agricultural University, Changsha 410128, China; e Yuelu Mountain Laboratory of Hunan Province, Changsha 410004, China

**Keywords:** Camellia seed cake, Acyl-CoA oxidase 1, Natural extract, Glucose and lipid metabolism, Type 2 diabetes

## Abstract

Acyl-CoA oxidase 1 (ACOX1) plays a key role in glycolipid metabolism disorders. Camellia seed cake, a byproduct of oil production, was identified as a source of ACOX1 inhibitors. Under optimized conditions (58 % ethanol, 70 °C, 62 min), the extract exhibited an 83.55 % inhibition rate and an IC_50_ of 18.88 mg/mL after purification. Among 138 compounds identified in the extract, 57 were flavonoids, with luteolin-4’-*O*-glucoside showing the highest binding affinity to ACOX1. In diabetic mice, the extract significantly reduced hepatic ACOX1 activity by 46.49 % and blood glucose levels by 25.76 %, and simultaneously decreased blood lipids and alleviated hepatic lipid accumulation. Oxidative stress was mitigated through reduced H₂O₂ production and enhanced antioxidant enzyme activity. Furthermore, ACOX1 inhibition lowered the hepatic NADH/NAD^+^ ratio by 34.96 %, thereby upregulating SIRT1 expression by 20.00 % and suppressing UCP2 by 33.04 %, ultimately increasing ATP levels by 14.66 %. Collectively, camellia seed cake extract ameliorates glycolipid metabolism disorders via ACOX1 inhibition.

## Highlights

• Extraction of ACOX1 inhibitors from camellia seed cake.
• ACOX1 inhibitors ameliorate glycolipid metabolism disorder in mice.
• ACOX1 inhibitors reduce oxidative stress in diabetic mice.
• ACOX1 inhibitors increase ATP level through the SIRT1-UCP2 axis.

## 1 Introduction

*Camellia oleifera* Abel., a small tree or shrub belonging to the family Theaceae and genus *Camellia*, is native to southern China and is one of the four major woody oil crops globally (Li et al., 2018). Camellia seed cake, with an annual production of 1.97 million tons, is the major by-product of camellia seed oil extraction (Hong et al., 2019). Camellia seed cake is rich in bioactive compounds such as flavonoids, saponins, polysaccharides, and proteins, which exhibit a variety of bioactivities (Liu & Wang, 2025). Saponins from *C. oleifera* exhibit anticancer activity and antioxidant activities (Di et al., 2017; Wang et al., 2025). Proteins and their hydrolysates display anticancer and antioxidant effects (Li et al., 2015; Li et al., 2019). Polysaccharides exhibit hypoglycemic and antioxidant activities (Zhang & Li, 2015; Xu et al., 2016; Jin et al., 2019). Flavonoids confer antioxidant and anti-inflammatory functions (Ye et al., 2012; Zheng et al., 2019). However, most of the camellia seed cake is currently undervalued, remains underutilized and simply discarded as industrial waste (Xie et al., 2023). Therefore, extracting its bioactive components from camellia seed cake and elucidating their mechanisms of action are crucial for unlocking its untapped potential.

Acyl-CoA oxidase 1 (ACOX1), the rate-limiting enzyme in peroxisomal fatty acid β-oxidation, is central to lipid metabolism and energy homeostasis (Ding et al., 2021; Zhang et al., 2023). Inhibition of ACOX1 activity by herbal formula or chemical molecules has been shown to reduce hepatic lipid accumulation, improve insulin sensitivity, and alleviate oxidative stress, making it a promising strategy for treating metabolic diseases (Shang et al., 2024; Zeng et al., 2017). Liver-specific knockout of ACOX1 has been reported to promote resistance to diet-induced obesity, inflammation, and insulin resistance (Lu et al., 2024). Although the herbal formula Shenge have demonstrated inhibitory effects on ACOX1 activity (Shang et al., 2024), their multi-component complexity obscures mechanistic interpretation and hinders industrial scalability. Single-plant-derived compounds, such as total glucosides of *Picrorhizae Rhizome*, saikosaponins A and D, schisandrin B, gentiopicroside, can modulate ACOX1 expression or protein stability (Zhuo et al., 2024; Li et al., 2021; Yan et al., 2022; Huang et al., 2024;). However, direct inhibition of ACOX1 by isolated phytochemicals from individual plant sources remains unreported to date.

This study aims to extract ACOX1 inhibitors from camellia seed cake. The key components responsible for ACOX1 inhibition in this plant resource will be identified. Animal experiments will be conducted to explore how camellia seed cake extracts ameliorate glycolipid metabolism disorder in mice by inhibiting ACOX1 activity.

## 2 Materials and methods

### 2.1 Chemicals and reagents

The camellia seed cakes were provided by the Hunan Academy of Forestry (Hunan, China). AB-8 macroporous resin was purchased from Tianjin Nankai Hecheng Technology Co., Ltd. (Tianjin, China). Streptozotocin (STZ) was obtained from Meilun Biotech (Shanghai, China). The total RNA extraction kit was sourced from Bioteke Corporation (Zhejiang, China). PrimeScript™ RT Reagent Kit and TB Green® Premix Ex Taq™ II were purchased from Takara Bio Inc. (Dalian, China). Primers were synthesized by Sangon Biotech (Shanghai, China). Anti-β-actin and Anti-Uncoupling Protein 2 (UCP2) antibodies were obtained from Cell Signaling Technology (Danvers, MA, USA). Anti-Silent Information Regulator 1 (SIRT1) and Anti-ACOX1 antibodies were obtained from Abcam (Cambridge, UK). All other reagents were of analytical grade and were purchased locally in China.

### 2.2 Extraction of ACOX1 inhibitors from camellia seed cake

The camellia seed cake was dried to a moisture content of 2 %, ground, and sieved through a 60-mesh sieve. The sample was defatted by ultrasonic-assisted extraction (300 W, 25 °C, 30 min) with n-hexane at a solid-to-liquid ratio of 1:3. After centrifugation (25 °C, 4000 ×*g*, 10 min), the supernatant was discarded, and the process was repeated four times to ensure thorough defatting. ACOX1 inhibitory activity was measured to evaluate the effects of extraction temperature (30–80 °C), ethanol concentration (30–90 %, *v*/v), solid-to-liquid ratio (1:2–1:12), and extraction time (10–100 min) on extraction efficiency in single-factor experiments. The initial extraction conditions were set at 30 °C, 50 % ethanol (*v*/v), a solid-to-liquid ratio of 1:10, and an extraction time of 30 min, with ultrasonic power fixed at 300 W.

Based on the results of the single-factor experiments, a Box-Behnken design was implemented using Design Expert 13 software (Stat-Ease, Inc., Minneapolis, MN, USA). The design optimized ethanol concentration, extraction temperature, and extraction time in a three-factor three-level response surface model, using ACOX1 inhibition rate as the response variable (**Table S1**). The model significance was assessed by Analysis of Variance (ANOVA), and the interactions were analyzed using response surface and contour plots to predict the optimal extraction conditions. The predicted results were then validated through verification experiments.

### 2.3 ACOX1 activity assay

The recombinant strain for ACOX1 production was preserved in this lab. The expression and purification of the recombinant ACOX1 were based on protocols described in previously published studies (Zeng & Li, 2004; Zeng et al., 2017). The liver peroxisomes were isolated for ACOX1 activity assay as described before (Zeng et al., 2017).

The ACOX1 activity was determined by monitoring the increase in absorbance at 367 nm, which corresponds to the formation of trans-3-indoleacryloyl-CoA (ε_367 nm_ = 26.5 mM^−1^ cm^−1^) from the substrate 3-indolepropionyl-CoA. The enzyme activity assay was performed in a reaction system containing 50 mM phosphate buffer (pH 7.4), 200 μg of recombinant ACOX1, 30 μM flavin adenine dinucleotide (FAD) (Sigma), and 0.02 % Triton X-100 (*w*/*v*), in a total volume of 500 μL. The reaction was initiated by adding 50 μM 3-indolepropionyl-CoA, and absorbance changes were measured using a Shimadzu UV-1800 UV–visible spectrophotometer. The ACOX1 inhibition rate was calculated using the following formula:

Inhibition rate (%) = [(A - B) / A] × 100 %, where A and B represent the ACOX1 activity without and with the extract, respectively.

### 2.4 Purification of ACOX1 inhibitors

The AB-8 macroporous resin was pretreated by soaking in 95 % ethanol (*v*/v) for 24 h, followed by repeated rinses with distilled water until the filtrate became clear. The resin was then soaked sequentially in 4 % NaOH (w/v) for 24 h and in 4 % HCl (w/v) for 24 h. After each treatment, it was rinsed with distilled water until the effluent reached pH 7.0, and stored in ultrapure water for future use.

AB-8 macroporous resin (20 g) was packed into a chromatography column and equilibrated with ultrapure water at a flow rate of 1 bed volume per hour (BV/h). The camellia seed cake extract was dissolved in ultrapure water (30 mg/mL), with pH adjusted to 3. The loading concentration and pH value were determined in pre-experiments as shown in **Fig. S1**. 0.5 BV of the extract solution was slowly loaded into the column at a flow rate of 0.5 BV/h. After loading, the column was eluted sequentially with 2 BV of ultrapure water, followed by a stepwise ethanol gradient (2 BV each of 20 %, 40 %, 60 %, 80 %, 100 % *v*/v) at 1 BV/h. The 40 % ethanol-eluted fraction was collected, concentrated by rotary evaporation (40 °C), and lyophilized to a powder.

Sample solutions with gradient concentrations of 1, 2, 4, 8, 16, 32, 64, 80, 96, 112, and 128 mg/mL were tested for ACOX1 inhibitory activity. The IC_50_ value was calculated using GraphPad Prism 8.0.2 based on the inhibition rate-concentration curve.

### 2.5 UHPLC-QE-Orbitrap-MS analysis of the camellia seed cake extract

Ultra-High Performance Liquid Chromatography-Quadrupole-Orbitrap Mass Spectrometry (UHPLC-QE-Orbitrap-MS) was employed for the analysis of purified extract. The sample was finely ground, and 50 mg was precisely weighed. Then, 500 μL of methanol:acetonitrile (1:1, v/v) mixture was added for dissolution, followed by sonication in an ice bath for 30 min. Afterward, the mixture was centrifuged at 20 °C, 17000 ×*g* for 15 min, and the supernatant was transferred to a new 1.5 mL microcentrifuge tube, discarding the pellet. The solvent was removed by vacuum evaporation at 35 °C, and the residue was reconstituted in 100 μL of 50 % (*v*/v) methanol. The solution was vortexed thoroughly and centrifuged again at 4 °C, 17000 ×g for 15 min.

The analysis was performed on a Thermo UltiMate 3000 UHPLC system equipped with a Waters HSS T3 column (100 × 2.1 mm, 1.8 μm). The column temperature was maintained at 40 °C, and the injection volume was 5 μL, with a flow rate of 0.4 mL/min. Samples were stored at 4 °C in the autosampler. The mobile phase consisted of 0.1 % formic acid in water (A) and 0.1 % formic acid in acetonitrile (B), using the following linear gradient program as described in previous report with minor modifications (Huang et al., 2018): 0–1.5 min, 95 % A; 1.5–2.5 min, 95 % to 90 % A; 2.5–14 min, 90 % to 60 % A; 14–22 min, 60 % to 5 % A; 22–23 min, 5 % A; 23–23.1 min, 5 % to 95 % A; 23.1–30 min, 95 % A.

For mass spectrometric analysis, an AB Sciex X500R Triple TOF mass spectrometer was employed to collect both MS1 and MS2 data. During each data acquisition cycle, the strongest ions with intensities greater than 100 were selected for MS/MS analysis. The MS1 acquisition range was 50–1200 *m*/*z*, with a collision energy of 30 eV and 10 MS2 spectra collected every 50 ms. The ESI ion source parameters were as follows: nebulizer gas pressure (GS1) at 60 psi, auxiliary gas pressure at 60 psi, curtain gas pressure at 35 psi, ion source temperature at 65 °C, and spray voltage at 5000 V.

The raw data was processed using ProteoWizard software to convert it into mzML format. Peak identification, filtration, and alignment were performed using MS-DIAL to generate a data matrix with information on mass-to-charge ratio (m/z), retention time, and intensity. Compound identification was initially identified by accurate molecular mass (with a mass error ≤ 30 ppm), and further confirmed by MS2 spectral databases (MassBank, GNPS, RIKEN PlaSMA, BMDMS-NP, and mzCloud) with a minimum match score of 80 to ensure identification reliability.

### 2.6 Molecular docking and molecular dynamics simulations

The crystal structure of mouse ACOX1 was predicted using AlphaFold (https://www.alphafold.ebi.ac.uk). Two-dimensional structures of bioactive compounds were retrieved from PubChem (http://pubchem.ncbi.nlm.nih.gov). The top 20 compounds identified in the camellia seed cake extract were converted into energy-minimized three-dimensional structures using Chem3D and docked to ACOX1. The ACOX1 protein was prepared using the Protein Preparation Wizard in Maestro 13.4 (Schrödinger, LLC, New York, NY, USA) to ensure it adopted the optimal conformation for docking. Docking was performed using the Glide program with the OPLS4 force field, following default parameters. Ligand-receptor binding modes were evaluated by free energy calculations via Molecular Mechanics/Generalized Born Surface Area (MM/GBSA) method. The binding modes and interactions between the ligands and receptor were visualized using Discovery Studio 4.5 Client (BIOVIA, San Diego, California, USA) .

Molecular dynamics simulations of the ACOX1-ligand complex were performed with GROMACS 2023. The CHARMM36 force field was used for the protein (Jo et al., 2008), and the ligand topology was constructed using the CGENFF force field parameters. The system was solvated in a cubic box with the TIP3P water model (Mark & Nilsson, 2001), and periodic boundary conditions were applied. Electrostatic interactions were treated using Particle Mesh Ewald (PME), and the Verlet algorithm was used for integration. The system was equilibrated for 100 ps using both NVT and NPT ensembles, with a coupling constant of 0.1 ps. Van der Waals and Coulombic interactions were calculated with a 1.0 nm cutoff. The final production run was carried out for 100 ns at constant temperature (300*K*) and pressure (1 bar).

### 2.7 Animal experiment

The experiment was conducted using 28 male 6-week-old KM mice, provided by Hunan Slyk Jingda Laboratory Animal Co., Ltd. (Hunan, China). Standard rodent chow (12 % of calories from fat) and high-fat diet containing 10 % (*w*/w) lard were also supplied by the same vendor. The mice were housed individually in cages with free access to food and water, under controlled temperature (22 °C) and light conditions (12 h light/12 h dark). After one week of acclimatization to a standard diet, the mice were randomly divided into two groups: normal control group (Group N), and the high-fat diet group. After being fed high-fat diet for 4 weeks, the mice in the high-fat diet group were treated with daily intraperitoneal injection of 40 mg/kg streptozotocin (STZ) for 7 consecutive days (Furman, 2015). After the completion of the STZ injections, blood glucose was monitored every week by tail vein sampling using a glucometer, with successful model induction defined as fasting blood glucose consistently exceeding 7 mmol/L. The diabetic mice were randomly divided into three groups: high-fat model group (Group M), low-dose extract treatment group (Group L), and high-dose extract treatment group (Group H). The mice in the N and M groups were orally administered sterile saline; those in the L group were given 250 mg/kg/d camellia seed cake extract; and those in the H group were given 1000 mg/kg/d camellia seed cake extract. Throughout the treatment period, the mice in the N group were fed standard rodent chow, while the remaining groups were provided with high-fat diet.

After 6 weeks of oral treatment, the mice were fasted for 12 h (with free access to water). The mice were anesthetized using sodium pentobarbital at a dose of 50 mg/kg (i.p.) and euthanized. Blood was collected via retro-orbital bleeding, left to clot at 25 °C for 2–3 h, and then centrifuged (25 °C, 4000 ×*g*, 10 min) to obtain serum for further analysis. Following blood collection, the liver was excised, washed with saline to remove blood, dried, and weighed. A small portion of liver tissue was placed in a sterile EP tube, snap-frozen in liquid nitrogen, and stored at −80 °C. Body weight, food intake, liver weight, and liver index (liver weight/body weight × 100 %) were recorded for each mouse.

Animal experiments were approved by the Institutional Animal Protection and Use Committee of Hunan Agricultural University (Ethics Certificate number, 2021–2138). The experimental procedures complied with the guide for the Care and Use of Laboratory Animals in the “Regulations for the Administration of Experimental Animals” and “Measures for the Administration of Experimental Animals in Hunan Province”. All efforts were made to ensure the welfare and ethical treatment of the animals throughout the study.

### 2.8 Oil red O staining

Liver tissue samples were subjected to Oil Red O staining to assess hepatic lipid accumulation. Frozen liver samples were sectioned into 10–15 μm slices and dried at 28 °C for 15 min. The sections were then washed in 60 % isopropanol for 5 min. Next, the tissue sections were stained with Oil Red O solution for 10 min. After staining, the sections were differentiated in 60 % isopropanol until the tissue boundaries became clearly visible. After three washes with ultrapure water, hematoxylin was applied for 2 min, followed by 2–3 washes with ultrapure water. The sections were then differentiated for approximately 2 s in 1 % hydrochloric acid alcohol (*w*/*v*). Finally, the slides were washed in ultrapure water for 10 min until they regained a blue color, dried, and mounted with glycerol gelatin.

### 2.9 Biochemical analysis

Serum glucose, insulin, free fatty acid (FFA) levels, as well as triglyceride (TG) and total cholesterol content (TC) in both serum and liver, liver total protein, hydrogen peroxide (H_2_O_2_) content, catalase (CAT) activity, adenosine triphosphate (ATP) content, malondialdehyde (MDA) levels, glutathione peroxidase (GSH-Px) activity, total superoxide dismutase (T-SOD) activity, and the ratio of reduced/oxidized nicotinamide-adenine dinucleotide (NADH/NAD^+^) in liver tissue were measured using commercially available kits (Nanjing Jiancheng Bioengineering Institute, Jiangsu, China). All assays were performed according to the manufacturer's instructions.

### 2.10 Quantitative real-time PCR

Total RNA was extracted from liver tissue using the BioFast SimplyP Total RNA Extraction Kit (Bioflux Bio, Zhenjiang, China). Reverse transcription was performed using the PrimeScript™ RT Reagent Kit (TaKaRa, Dalian, China), and quantitative PCR (qPCR) was conducted with the TB Green® Premix Ex Taq Kit (TaKaRa, Dalian, China). The relative expression levels of target genes were normalized to *β-actin* as the internal reference gene, and were calculated using the 2^−ΔΔCT^ method. Primer sequences are listed in **Table S2**.

### 2.11 Western blot

During the experiment, the liver tissue (30 mg) was minced and homogenized in 0.3 mL ice-cold RIPA buffer containing a protease inhibitor. In the detection process, the protein concentration was quantified using a bicinchoninic acid (BCA) protein assay kit (Beyotime Biotechnology, Shanghai, China). An equal amount of protein samples was separated by 10 % SDS-PAGE gels, transferred to a nitrocellulose membrane, and blocked with 5 % bovine serum albumin (BSA) in TBST for 1 h. Then, the protein samples were incubated in primary antibody against SIRT1 (ab189494, Abcam, Cambridge, MA, USA; 1:1000), ACOX1 (ab184032, Abcam, Cambridge, MA, USA; 1:1000), UCP2 (89,326, Cell Signaling Technology, Danvers, MA, USA, 1:1000), and β-actin (4970S, Cell Signaling Technology, Danvers, MA, USA, 11000) in 3 % (*w*/*v*) BSA at 4 °C overnight, rinsed with TBST (Tris buffered saline, 0.1 % Tween 20), and subsequently incubated in HRP-conjugated secondary antibody (HRP) (7074S, Abcam, Cambridge, MA, USA, 15000) at room temperature for 2 h. The PVDF membrane was immersed in enhanced chemiluminescence (ECL) reagent (Beyotime Biotechnology Co., Ltd., Shanghai, China) for 5 min. After removal, the membrane was imaged using a gel imaging system. The intensities of the electrophoretic bands were analyzed using ImageJ software, and the relative protein levels in each group were normalized to β-actin.

### 2.12 Data processing and analysis

Statistical analysis and data visualization were performed using GraphPad Prism 8.0.2 (GraphPad Software, San Diego, California, USA). Comparisons between two groups were conducted using *t*-test, and one-way analysis of variance (ANOVA) was used for comparisons among multiple groups. A *P* value of <0.05 was considered statistically significant, and *P* < 0.01, *P* < 0.001, and *P* < 0.0001 were regarded as highly significant.

## 3 Results

### 3.1 Extraction of ACOX1 inhibitor from camellia seed cake

**Fig. 1:**
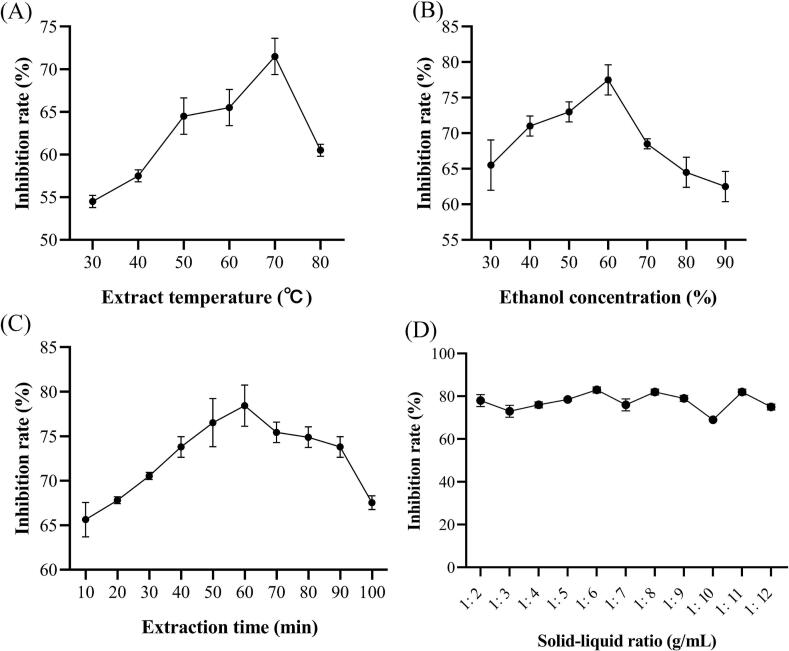
Results of single-factor optimization experiments for extracting ACOX1 inhibitors from camellia seed cake. (A) Effect of extraction temperature, (B) Effect of ethanol concentration, (C) Effect of extraction time, (D) Effect of solid-liquid ratio.

Extraction of ACOX1 inhibitors from camellia seed cake was optimized using the response surface methodology, with ACOX1 inhibition rate as the response variable. A series of single-factor experiments were conducted to examine the effects of extraction temperature, ethanol concentration, extraction time, and solid-liquid ratio (Fig. 1A-D). Fig. 1A shows that the inhibition rate increases with extraction temperature when it is below 70 °C, peaking at 71.60 % (70 °C) before declining. Fig. 1B indicates that maximal inhibition (77.50 %) at an ethanol concentration of 60 %, but decreases as the concentration continues to rise. Fig. 1C demonstrates that the inhibition rate reaches a maximum of 78.44 % at an extraction time of 60 min, followed by a gradual decline. Fig. 1D reveals that the solid-liquid ratio has minimal impact on the inhibition rate. The solid-liquid ratio was therefore fixed at 1:6 for further optimization.

The results of optimizing the extraction process of ACOX1 inhibitors using response surface methodology are presented in **Table S3**. The model was statistically significant (*P* < 0.01), with a non-significant lack of fit (*P* = 0.0979). The coefficient of determination (R^2^) of 0.9114 indicated a good model fit, and the adjusted coefficient of determination (R^2^ Adj) was 0.7975. In addition, the coefficient of variation (CV) was relatively small at 2.74 %, indicating that the model reflects the relationship between the ACOX1 inhibition rate of camellia seed cake extracts and factors such as ethanol concentration, extraction temperature, and extraction time. These results support the reliability of this experimental method (**Table S4**). The second-order regression model derived from the data was:

$$Y=84.27–0.68\;A-0.24B+1.47C+2.31\mathrm{AB}-2.39\;\mathrm{AC}+3.42\;\mathrm{BCE}-3.04A^{2}–2.80B^{2}–5.59C^{2}$$

**Fig. 2:**
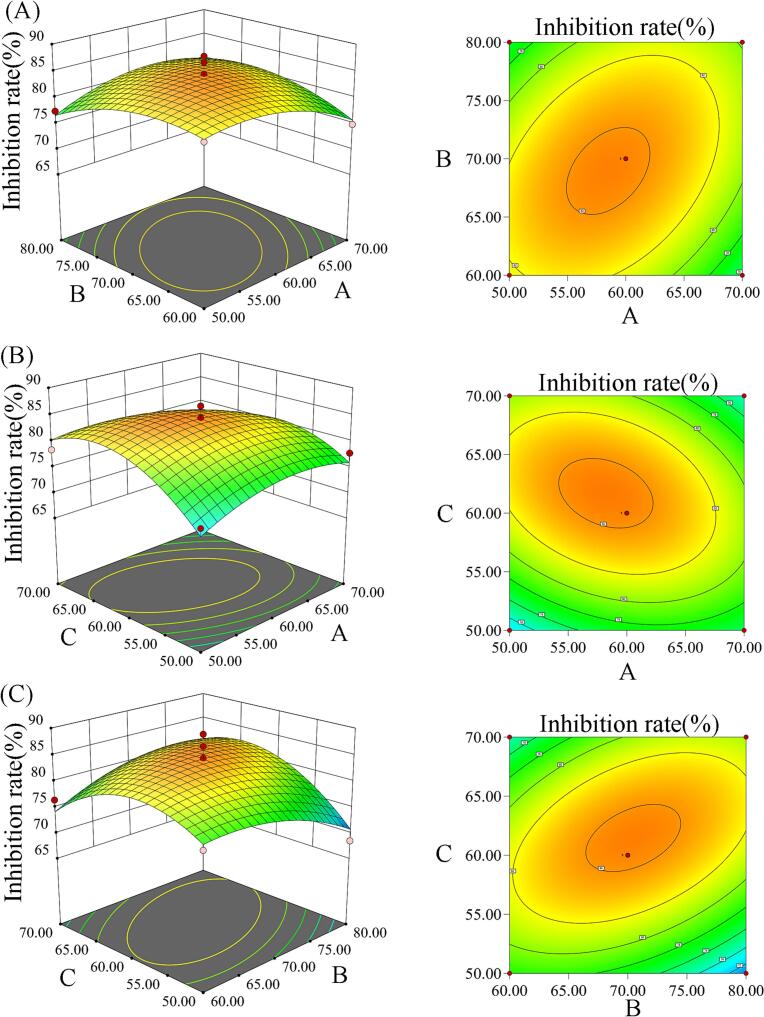
Response surface analysis of the effects of ethanol concentration, extraction temperature, and extraction time on the ACOX1 inhibitory rate. (A) Ethanol concentration and extraction temperature (A: ethanol concentration, %; B: extraction temperature, °C), (B) Ethanol concentration and extraction time (A: ethanol concentration, %; C: extraction time, min), (C) Extraction temperature and extraction time (B: extraction temperature, °C; C: extraction time, min).

The regression coefficients indicate that extraction time is the most influential factor affecting the ACOX1 inhibition rate, followed by ethanol concentration. Extraction temperature, in contrast, had a minor impact. Interactions among these factors were further assessed using three-dimensional response surface plots and contour plots (Fig. 2A-C). These analyses revealed that extraction time and ethanol concentration had the most significant influence on the inhibition rate.

Based on the regression model, the optimal extraction conditions were 58.17 % ethanol (*v*/v), 69.84 °C, and 61.66 min, with a predicted ACOX1 inhibition rate of 84.45 %. To facilitate practical application, the extraction parameters were adjusted to 58 % ethanol (v/v), 70 °C, and 62 min. Under these optimized conditions, the experimental ACOX1 inhibition rate was 83.55 ± 0.30 %, closely matching the predicted value and validating the reliability of the model. The extract was purified using macroporous resin chromatography, and the obtained fractions were used in subsequent experiments. After purification, the IC_50_ of the camellia seed cake extract against ACOX1 decreased from 36.62 mg/mL to 18.88 mg/mL, indicating a 48.44 % decrease (**Fig. S2**).

### 3.2 Analysis of camellia seed cake extract using UHPLC-QE-Orbitrap-MS

The camellia seed cake extract was analyzed using UHPLC-QE-Orbitrap-MS. The total ion chromatogram (TIC) in positive ion mode is shown in **Fig. S3**. A total of 138 chemical constituents were identified based on their accurate molecular weights, fragment ion information, and database comparisons (**Table S5**). Flavonoids were the predominant compounds, with 57 species identified, accounted for 84.80 % of total peak area, including kaempferol, kaempferol-3-*O*-rutinoside, and luteolin-4’-*O*-glucoside. These results indicate that the camellia seed cake extract is primarily composed of flavonoids, which exhibit significant structural diversity, providing a chemical foundation for further studies on its functions and bioactivity.

### 3.3 Molecular docking and molecular dynamics simulation analysis

Among the top 20 most abundant compounds identified from the camellia seed cake extract, five compounds with strong binding affinity to ACOX1 were selected based on their Docking Score (<−7 kJ/mol) and estimated Binding Free Energy (ΔG bind) values with MM/GBSA (<−34 kJ/mol) (**Table S6**). These compounds include luteolin-7-*O*-glucoside, luteolin-4’-*O*-glucoside, kaempferol, kaempferol-3-*O*-galactoside-7-*O*-rhamnoside and 5,7-dihydroxy-2-(4-hydroxyphenyl)-4H-chromen-4-one.

**Fig. 3:**
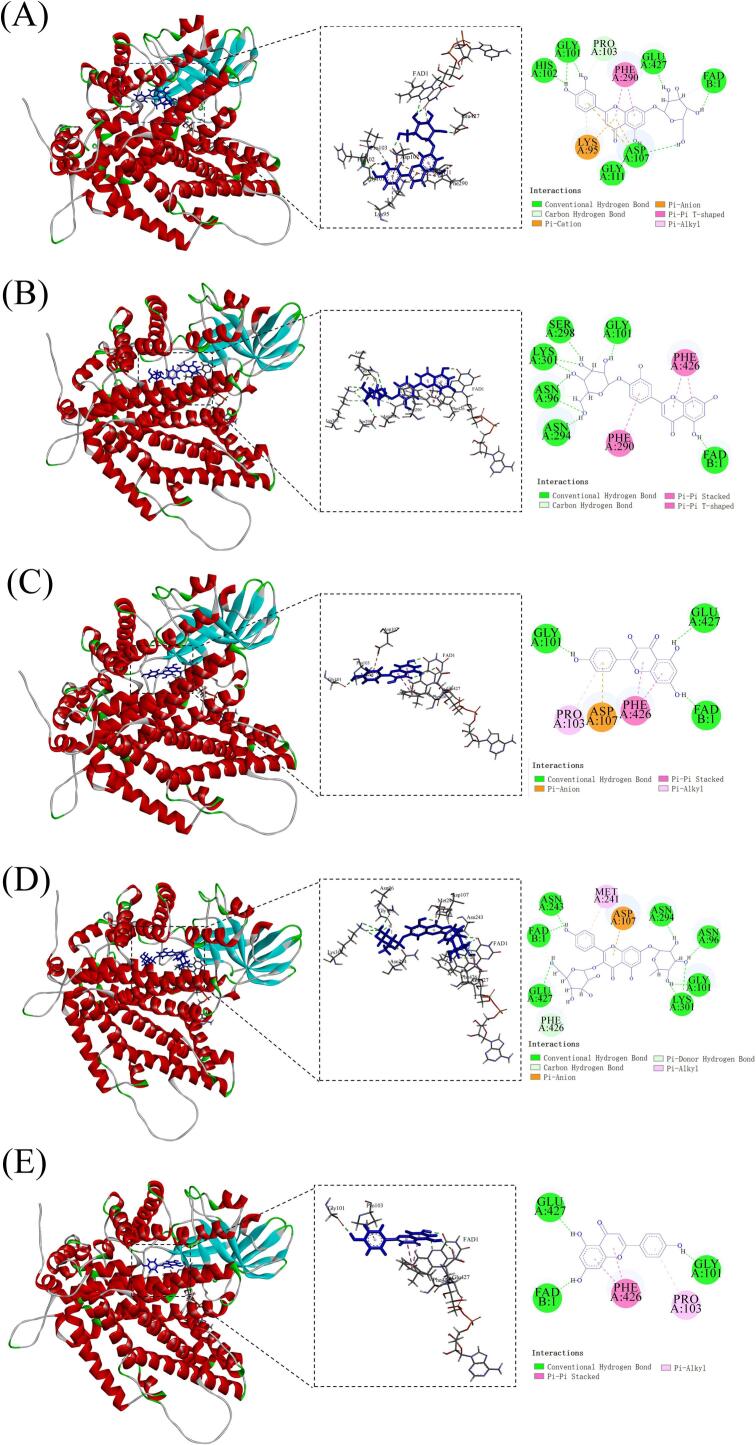
Molecular docking results of five compounds from camellia seed cake extract with ACOX1. 3D and 2D ligand interactions between ACOX1 and (A) Luteolin-7-*O*-glucoside, (B) Luteolin-4’-*O*-glucoside, (C) Kaempferol, (D) Kaempferol-3-*O*-galactoside-7-*O*-rhamnoside, (E) 5,7-dihydroxy-2-(4-hydroxyphenyl)-4H-chromen-4-one.

Luteolin-7-*O*-glucoside formed ten hydrogen bonds with residues Gly-101, His-102, Asp-107, Glu-427, Pro-103, Gly-111 and FAD. It also engaged in hydrophobic interactions with residues Lys-95 (Pi-Alkyl), Phe-290 (Pi-Pi T-shaped), and electrostatic interactions with residues Asp-107 (Pi-Anion and Pi-Cation), Lys-95 (Pi-Anion) (Fig. 3A). Luteolin-4’-*O*-glucoside formed eleven hydrogen bonds with residues Asn-96, Gly-101, Asn-294, Ser-298, Lys-301 and FAD. Additionally, it engaged in hydrophobic interactions with residues Phe-290 (Pi-Pi T-shaped), Phe-426 (Pi-Pi Stacked) (Fig. 3B). Kaempferol formed three hydrogen bonds with residues Gly-101, Glu-427 and FAD, as well as hydrophobic interactions with residues Pro-103 (Pi-Alkyl), Phe-426 (Pi-Pi Stacked), and electrostatic interactions with residues Asp-107 (Pi-Anion) (Fig. 3C). Kaempferol-3-*O*-galactoside-7-*O*-rhamnoside formed fourteen hydrogen bonds with residues Asn-96, Gly-101, Asn-243, Asn-294, Lys-301, Glu-427, Phe-426 and FAD. Furthermore, it exhibited a hydrophobic interaction with Met-241 (Pi-Alkyl), and electrostatic interactions with residues Asp-107 (Pi-Anion) (Fig. 3D). 5,7-dihydroxy-2-(4-hydroxyphenyl)-4H-chromen-4-one formed three hydrogen bonds with residues Gly-101, Glu-427 and FAD. It also engaged in hydrophobic interactions with residues Pro-103 (Pi-Alkyl) and Phe-426 (Pi-Pi Stacked) (Fig. 3E).

**Fig. 4:**
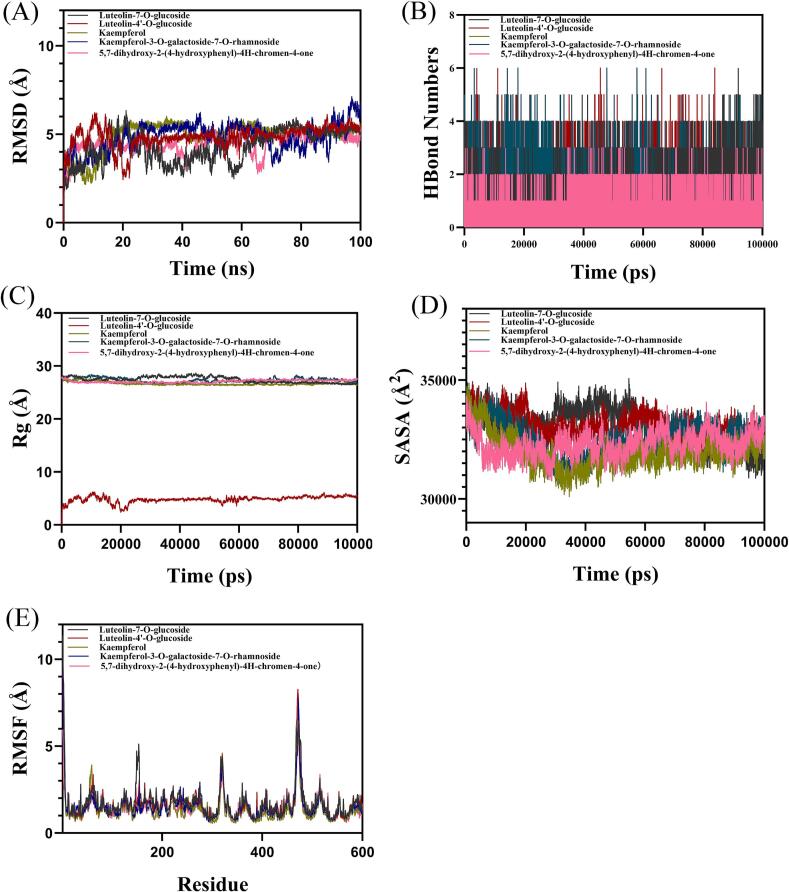
Molecular dynamics simulation of compounds in complex with ACOX1. (A) RMSD values, (B) HBonds Numbers, (C) Rg values, (D) SASA values, (E) RMSF values.

Molecular dynamics simulations were conducted to assess the stability and interaction characteristics of the five compounds (luteolin-7-*O*-glucoside, luteolin-4’-*O*-glucoside, kaempferol, kaempferol-3-*O*-galactoside-7-*O*-rhamnoside, and 5,7-dihydroxy-2-(4-hydroxyphenyl)-4H-chromen-4-one) in complex with ACOX1. Key parameters such as root mean square deviation (RMSD), radius of gyration (Rg), solvent accessible surface area (SASA), hydrogen bonds (HBonds), and root mean square fluctuation (RMSF) were analyzed to evaluate the complex stability. As shown in Fig. 4 **A**, the ACOX1-luteolin-4’-*O*-glucoside and ACOX1-kaempferol complexes reached equilibrium after 25 ns, with fluctuations around 5 Å. In contrast, the other three complexes reached equilibrium only after 60 ns. Hydrogen bonds play a critical role in ligand-protein binding. As shown in Fig. 4 **B**, the number of hydrogen bonds between ACOX1 and the ligands ranged from 0 to 6 for luteolin-4’-*O*-glucoside, luteolin-7-*O*-glucoside, and kaempferol-3-*O*-galactoside-7-*O*-rhamnoside, whereas the other two complexes exhibited hydrogen bond counts ranging from 0 to 3. Further analysis revealed that the Rg value of the ACOX1-luteolin-4’-*O*-glucoside complex remained below 10 Å and stabilized after 20,000 ps, while the SASA value slightly decreased after binding and then plateaued. The other four complexes maintained an Rg value around 29 Å. These findings indicate that the ACOX1-luteolin-4’-*O*-glucoside complex remained compact and stable throughout the simulation (Fig. 4 **C,D**). RMSF, which reflects the flexibility of amino acid residues, showed relatively low values (mostly below 4 Å) for all five complexes, indicating low flexibility and high stability. However, higher dynamic fluctuations were observed between residues 300–350 and 450–500 (Fig. 4 **E**).

In summary, the ACOX1-luteolin-4’-*O*-glucoside complex demonstrated the fastest RMSD equilibrium, the lowest Rg value, and the highest number of hydrogen bonds, indicating that luteolin-4’-*O*-glucoside binds effectively with ACOX1.

### 3.4 Effect of camellia seed cake extract on body weight, blood glucose, and lipid levels in diabetic mice

**Fig. 5:**
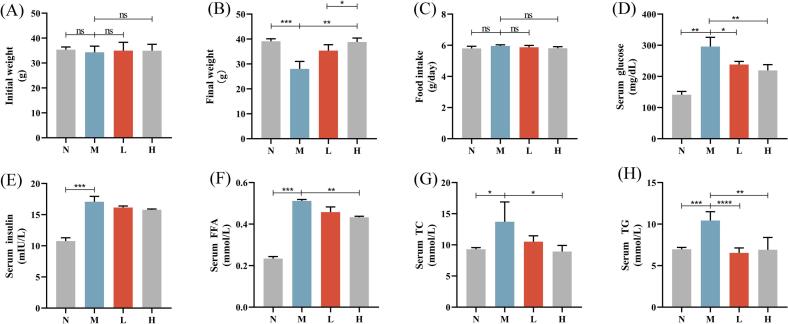
Camellia seed cake extract increased body weight and reduced serum glucose and lipid levels in diabetic mice. (A) Initial body weight, (B) Final body weight after the treatment, (C) Average food intake, (D) Serum glucose, (E) Serum insulin, (F) Serum FFA, (G) Serum TC, (H) Serum TG. *P < 0.05, **P < 0.01, ***P < 0.001,****P < 0.0001. Values are expressed as mean ± standard deviation (*n* = 7 per group).

To evaluate the effects of camellia seed cake extract on glucose and lipid metabolism, type 2 diabetic animal models were established via intraperitoneal injection of STZ. At baseline (day 0), body weights did not differ significantly among groups (*P* > 0.05, Fig. 5A). After oral treatment, M group showed significantly decreased body weight versus N group (*P* < 0.001). In contrast, H group mice showed significantly increased weight versus M group (*P* < 0.01, Fig. 5B). There was no significant difference in food intake among the groups (*P* > 0.05, Fig. 5C).

Compared to M group, treatment with the extract significantly reduced glycemia in L group (*P* < 0.05) and H group (*P* < 0.001, Fig. 5D), with dose-dependent effect (*P* < 0.05). Specifically, compared to the M group, blood glucose levels decreased by 19.32 % in L group and 25.76 % in H group. Although insulin levels in the L and H groups showed a decreasing trend, the difference was not statistically significant (Fig. 5E). Relative to M group, serum levels of FFA, TG and TC were significantly reduced in H group (*P* < 0.001 for FFA and TG, *P* < 0.01 for TC), while L group showed a significant reduction only in TG (*P* < 0.0001, Fig. 5F-H).

In summary, the camellia seed cake extract demonstrated beneficial effects on body weight, blood glucose, and blood lipids in diabetic mice.

### 3.5 Camellia seed cake extract alleviated hepatic lipid accumulation in diabetic mice

**Fig. 6:**
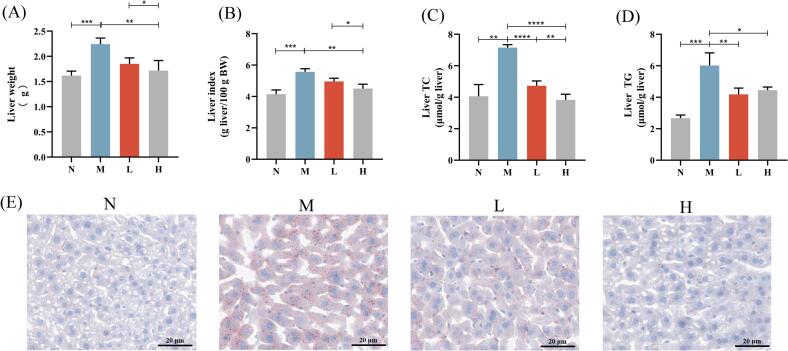
Camellia seed cake extract reduced lipid accumulation in the liver. (A) Liver weight, (B) Liver index, (C) Liver TC, (D) Liver TG, (E) Oil red O staining of liver tissues. Scale bar, 20 μm. * *P* < 0.05, ** *P* < 0.01, *** *P* < 0.001,**** *P* < 0.0001. Values are expressed as mean ± standard deviation (n = 7 per group). (For interpretation of the references to color in this figure legend, the reader is referred to the web version of this article.)

Treatment with camellia seed cake extract significantly reduced hepatic lipid accumulation in diabetic mice. Relative to M group, liver weight and index decreased significantly in H group (*P* < 0.01), while L group showed non-significant reduction (Fig. 6A and B). Hepatic TC decreased dose-dependently in L and H groups compared to M group (Fig. 6C). Hepatic TG declined significantly in L group (*P* < 0.01), while H group showed milder but still significant reduction (*P* < 0.05, Fig. 6D).

Oil Red O staining further confirmed reduced lipid accumulation. Compared to M group, L group exhibited fewer hepatic lipid droplets, while H group showed morphology similar to normal controls (Fig. 6E). These results indicate that camellia seed cake extract significantly reduces hepatic TC and TG levels and alleviates lipid deposition in a dose-dependent manner.

### 3.6 Camellia seed cake extract inhibits ACOX1, relieves hepatic oxidative stress, and improves energy metabolism in mice

**Fig. 7:**
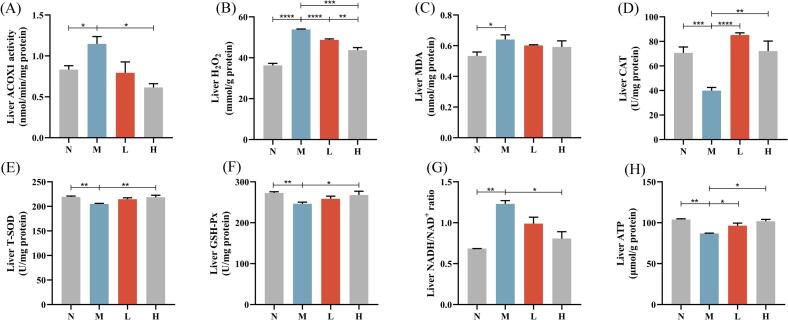
Inhibition of ACOX1 activity by camellia seed cake extract alleviates oxidative stress and improves energy metabolism. (A) Liver ACOX1 activity, (B) Liver H_2_O_2_ content, (C) Liver MDA content, (D) Liver CAT activity, (E) Liver T-SOD activity, (F) Liver GSH-Px activity, (G) Liver NADH/NAD^+^ ratio, (H) Liver ATP content. * *P* < 0.05, ** *P* < 0.01, *** *P* < 0.001,**** *P* < 0.0001. Values are expressed as mean ± standard deviation (n = 7 per group).

Camellia seed cake extract significantly improved hepatic antioxidant functions and energy metabolism in diabetic mice. Compared to M group, hepatic ACOX1 activity was suppressed in H group (*P* < 0.05). Specifically, the inhibition rate was 30.70 % in L group and 46.49 % in the H group (Fig. 7A). The inhibition rate of ACOX1 is directly proportional to the dose of the extract. Hepatic H₂O₂ was elevated in M group vs. N group (*P* < 0.0001). Both L and H groups showed a significant reduction in H_2_O_2_ levels compared to the M group (L group: *P* < 0.0001; H group: *P* < 0.001) (Fig. 7B).

MDA levels were significantly higher in M group vs. N group (*P* < 0.05), while both L and H groups showed a decrease in MDA levels, although this reduction was not statistically significant (Fig. 7C). Regarding antioxidant enzymes, CAT activity was significantly elevated in L (*P* < 0.001) and H groups (*P* < 0.01) vs. M group. Additionally, H group showed significantly enhanced GSH-Px (*P* < 0.05) and T-SOD (*P* < 0.01) activities (Fig. 7 **D—F**).

Correlation analysis in **Table S7** indicated significant positive correlations among hepatic antioxidant enzymes CAT, GSH-Px, and T-SOD (*P* < 0.05), suggesting their activities were simultaneously induced. Additionally, both GSH-Px and T-SOD exhibited statistically significant negative correlations with hepatic H₂O₂ and MDA levels. A strong positive correlation was observed between H₂O₂ and MDA content (*P* < 0.05). Conversely, CAT activity demonstrated non-significant negative correlations with both H₂O₂ and MDA (*P* > 0.05), potentially attributable to compensatory mechanisms from alternative antioxidant pathways.

NADH/NAD^+^ ratio was significantly higher in M group vs. N group (*P* < 0.01), while H group exhibited significant reduction vs. M group (*P* < 0.05, Fig. 7G). ATP levels were significantly lower in M group vs. N group (*P* < 0.01), whereas both L and H groups showed significant increases (L group: *P* < 0.05; H group: *P* < 0.01, Fig. 7H).

In conclusion, camellia seed cake extract effectively inhibits ACOX1 activity, restores redox homeostasis, alleviates oxidative stress, and improves hepatic energy metabolism.

### 3.7 Effects of camellia seed cake on hepatic lipid and glucose metabolism in type 2 diabetic mice

**Fig. 8:**
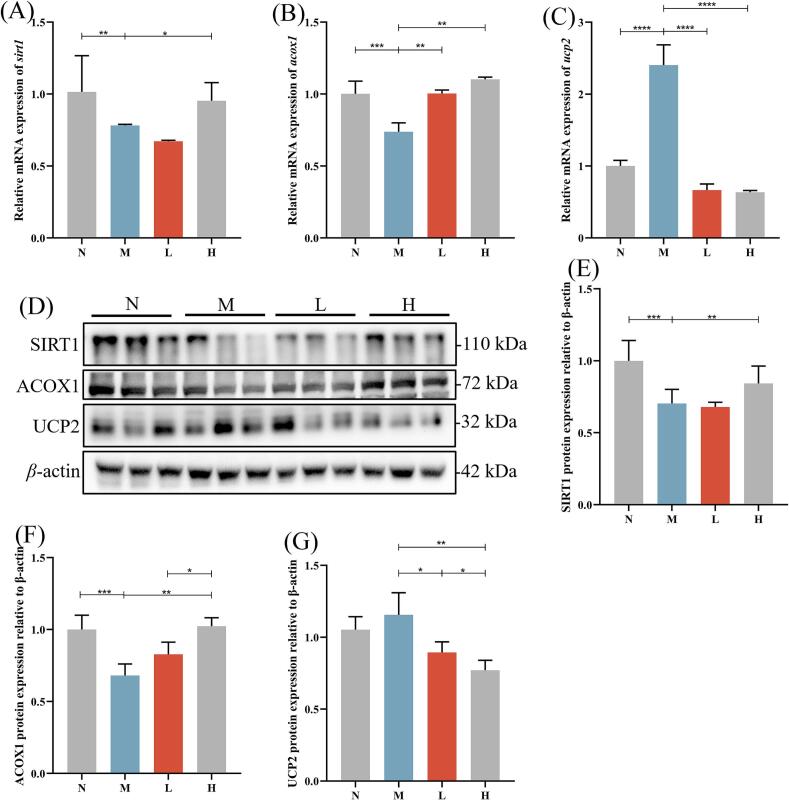
Effects of camellia seed cake extract on glucose and lipid metabolism in the liver of diabetic mice. Relative mRNA expression levels of genes analyzed by qPCR: (A) *sirt1*, (B) *acox1,* and (C) *ucp2*. (D) Representative Western blot analysis with β-actin as loading control. Quantified protein expression: (E) SIRT1, (F) ACOX1, and (G) UCP2. * *P* < 0.05, ** *P* < 0.01, *** *P* < 0.001,**** *P* < 0.0001.

As shown in Fig. 8, camellia seed cake extract significantly affected the levels of specific mRNAs and proteins in the liver of diabetic mice.

At the mRNA level, compared to N group, *acox1* expression was significantly downregulated in M group (*P* < 0.001). In contrast, *acox1* was significantly upregulated in L group (by 36.99 %) and H (by 50.68 %) group (*P* < 0.01). Similarly, the mRNA of *sirt1* was significantly reduced in M group (*P* < 0.01), while it was significantly increased in H group (*P* < 0.05). The *ucp2* expression was significantly elevated in M group vs. N group (*P* < 0.0001). In contrast, *ucp2* expression was significantly downregulated in both L and H groups (*P* < 0.0001) (Fig. 8A-C).

Western blot analysis (Fig. 8D) revealed that, at the protein level, expression of SIRT1 and ACOX1 was significantly suppressed in M group vs. N group (*P* < 0.001, Fig. 8E-G). Compared to M group, the ACOX1 protein expression level was upregulated by 20.59 % in the L group and by 50.00 % in the H group (Fig. 8F). Moreover, the H group exhibited significant upregulation of SIRT1 (*P* < 0.01) (Fig. 8E), while UCP2 was downregulated in both L (*P* < 0.05) and H groups (*P* < 0.01) (Fig. 8G).

## 4 Discussion

Existing studies demonstrate the potential of camellia products in regulating glucose and lipid metabolism. Our previous research revealed that virgin camellia seed oil improved high-fat diet-induced metabolic disorders in rats by activating adenosine 5′-monophosphate (AMP)-activated protein kinase (AMPK) and fatty acid oxidation enzymes, while suppressing lipid synthesis via downregulating sterol-regulatory element binding protein 2 (SREBP2) and its target genes (Zhu et al., 2023). These findings underscore the critical role of bioactive compounds in camellia seed in metabolic regulation. This study therefore focuses on camellia seed cake, an oil-extraction byproduct, to explore its bioactivities and metabolic effects. Camellia seed cake is rich in bioactive components, including polysaccharides, flavonoids, and triterpene saponins (Quan et al., 2022). Although annual production reached 1.97 million tons (Hong et al., 2019), its high-value utilization remains limited (Xie et al., 2023).

ACOX1 serves as the key enzyme initiating peroxisomal fatty acid β-oxidation, catalyzing the decomposition of long-chain acyl-CoA into shorter-chain fatty acids and hydrogen peroxide (Zhang et al., 2023). Its dysregulation is closely linked to various metabolic disorders. Previous study confers that the knockout of ACOX1 in mice exhibits significant protection against diet-induced obesity, adipose tissue inflammation, and systemic insulin resistance (Lu et al., 2024). Conversely, elevated ACOX1 expression in macrophages promotes pro-inflammatory phenotypes in type 1 diabetes, accelerating atherosclerosis progression (Kanter et al., 2012). These findings establish ACOX1 as a therapeutic target for metabolic syndrome, obesity, and diabetes.

Camellia seed cake was identified as a source of ACOX1 inhibitors in this study. The inhibition rate of the extract was optimized via response surface methodology, and further elevated through resin purification. UHPLC-QE-Orbitrap-MS analysis revealed that the purified fraction comprised mainly flavonoids. Flavonoids from camellia seed cake exhibit diverse health benefits, including antioxidative, anti-inflammatory, antiviral, and antimicrobial activities (Ye et al., 2012; Zheng et al., 2019; Li et al., 2014; Wei et al., 2014; Xiao, 2017), potentially preventing obesity, cardiovascular disorders, and diabetes (Gentile et al., 2018; Perez-Vizcaino & Fraga, 2018; Al-Ishaq et al., 2019). Their identification as ACOX1 inhibitors elucidates their mechanism in suppressing peroxisomal fatty acid β-oxidation, providing valuable insights for metabolic disease therapeutics.

Through molecular docking and molecular dynamics simulations, this study identified luteolin-4’-*O*-glucoside as the most potent ACOX1 binder. While direct evidence of its glycemic role is lacking, this study suggests its potential in modulating glucose and lipid metabolism via ACOX1 inhibition. Previous research has demonstrated similar effects for luteolin and luteolin-7-*O*-glucoside in glycemic control (Zang et al., 2016; Park & Song, 2013; Hu & Kitts, 2004; Johnson et al., 2021), supporting structural homology. We therefore propose luteolin-4’-*O*-glucoside as a candidate ACOX1 inhibitor and therapeutic agent for metabolic disorders, enabling exploration of flavonoid-based therapies.

In this study, camellia seed cake extract was orally administered to type 2 diabetic mice, and demonstrated therapeutic effects. Efficacy was verified through physiological measurements, with mechanisms elucidated via key gene/protein analyses. Our results showed *in vivo* ACOX1 inhibitory activities of 46.49 % at a dose of 1000 mg/kg/d. Shang et al. Reported approximately 50 % ACOX1 inhibition in high-fat diet-fed mice treated with the Shenge formula at a dose of 9 g/kg/d (Shang et al., 2024), a traditional Chinese medicine (TCM) prescription containing multiple herbs with clinical use in non-alcoholic liver disease (NAFLD). Although synergistic effects may exist in traditional Chinese medicine (TCM) formulas, their multi-component nature makes it difficult to definitively identify the botanical origin of key active compound monomers, thereby limiting subsequent optimization processes. Both interventions share ACOX1 inhibition mechanisms, but the purified extract achieved comparable efficacy via a much smaller dose, underscoring the extract's translational value in treating ACOX1-associated metabolic disorders.

Oxidative stress is a critical pathological mechanism in type 2 diabetes. Insulin resistance induces hepatic fatty acid oxidation and ACOX1 upregulation, elevating H_2_O_2_ production that exacerbates oxidative stress (Zakaria et al., 2017). Concurrently, dysfunctional antioxidant enzyme system (GSH-Px/T-SOD/CAT) impair H₂O₂ clearance, causing lipid peroxidation (e.g., MDA accumulation), membrane damage, and apoptosis (Coskun et al., 2005). Camellia seed cake extract inhibited ACOX1 (the key peroxisomal H₂O₂-producing enzyme), while coordinately upregulating antioxidant enzymes to alleviate oxidative stress damage. Furthermore, the extract reduced NADH/NAD^+^ ratio, increased ATP levels, and optimized hepatic energy metabolism, this is consistent with previous studies (Chen et al., 2020). We propose that improving energy metabolism is also part of the mechanism of action of ACOX inhibitors in camellia seed cake extract.

**Fig. 9:**
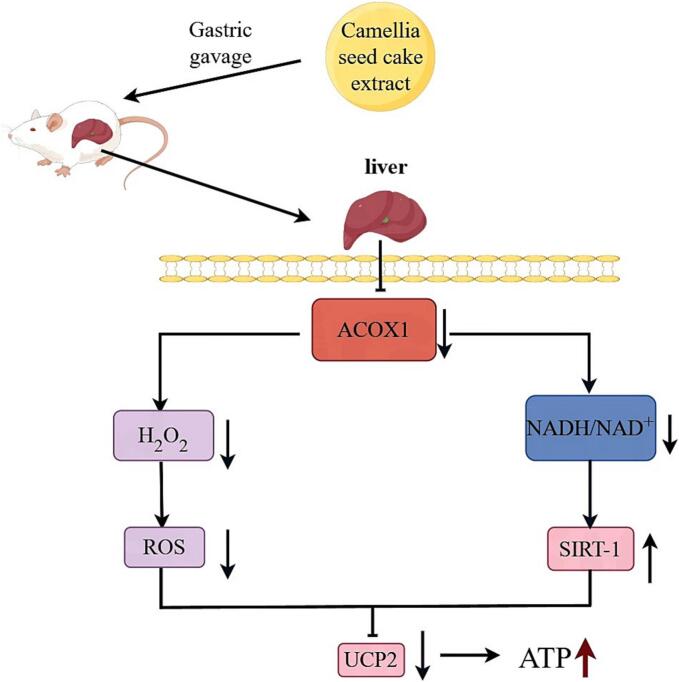
Mechanism by which camellia seed cake extract ameliorates glycolipid metabolism disorders in diabetic mice (diagram by Figdraw 2.0).

Through gene/protein analyses, this study elucidates how camellia seed cake extract regulates glucose/lipid metabolism via ACOX1 inhibition. Results show significantly elevated ACOX1 expression in M group. Despite extract-mediated ACOX1 inhibition, compensatory upregulation occurs via peroxisome proliferators-activated receptor alpha (PPARα) activation by accumulated substrates (Cantó & Auwerx, 2012). This inhibition reduces excessive production of H₂O₂ and NADH during peroxisomal fatty acid β-oxidation, thereby alleviating oxidative stress, lowering reactive oxygen species (ROS) levels, and suppressing UCP2 expression (Kizaki et al., 2002). Additionally, increased NAD^+^ levels activate SIRT1 (Tu et al., 2013). Activated SIRT1 transcriptionally downregulates UCP2 (Bordone et al., 2006). UCP2 gene polymorphisms are strongly associated with the development of diabetes (Gomathi et al., 2019). Knockout of UCP2 in pancreatic β-cells enhances glucose-induced insulin secretion (Robson-Doucette et al., 2011), while its overexpression inhibits this process, increasing the risk of type 2 diabetes in humans (Chan et al., 1999). We hypothesize that UCP2 uncoupling reduces ATP synthesis, decreases the ATP/ADP ratio, and thereby suppresses insulin secretion (Schumann et al., 2020; Sreedhar & Zhao, 2017). In conclusion, camellia seed cake ameliorates glucose and lipid metabolism disorders through dual mechanisms: (1) ACOX1 inhibition reduces oxidative stress and downregulates UCP2; (2) increased NAD^+^ activates SIRT1 to transcriptionally suppress UCP2. These findings provide a theoretical basis for developing ACOX1-targeted therapeutics against metabolic disorders (Fig. 9).

Dietary supplementation of ACOX1 inhibitor has the potential to be an effective strategy for treating obesity-associated metabolic disorders (Lu et al., 2024; Shang et al., 2024; Zeng et al., 2017). Although our findings highlight the therapeutic potential of camellia seed cake extract in improving glucose and lipid metabolism, several critical issues remain unresolved. Future research should focus on isolating and validating individual active components and investigating their specific mechanisms of action. Additionally, integration of high-throughput technologies such as metabolomics could provide deeper insights into the multidimensional regulatory pathways influenced by the extract. Lastly, evaluation of the interactions between the extract and existing antidiabetic drugs would help determine potential synergistic effects and clinical utility in managing metabolic syndrome. Regarding clinical translation, the present study does not evaluate the compound stability, human dosage, or long-term toxicity of the extract, which require further verification.

## 5 Conclusions

This study confirmed camellia seed cake as a resource for ACOX1 inhibitors, and systematically optimized the extraction and purification processes. Further analysis revealed that the extract was predominantly composed of flavonoids, among which molecules such as luteolin-4’-*O*-glucoside were identified as the key ACOX1 binders. The in vivo inhibitory activity of the extract was evaluated. The extract ameliorated weight loss, reduced blood glucose and lipid levels, and improved hepatic lipid accumulation and oxidative stress in diabetic mice. Mechanistically, by inhibiting ACOX1 to alleviate oxidative stress and elevating NAD^+^ to activate SIRT1, the extract synergistically downregulated UCP2, thereby restoring glucose-lipid metabolism and energy production. Although the extract demonstrates prominent metabolic regulatory effects, future toxicological evaluations and target validation are needed to ensure clinical translatability. The findings establish a mechanistic basis for camellia seed cake extract as a potential therapeutic agent for metabolic diseases.

## CRediT authorship contribution statement

**Bolin Chen:** Writing – original draft, Software, Formal analysis, Data curation. **Li Ma:** Visualization, Validation, Data curation. **Xinzhi Chen:** Writing – original draft, Validation, Methodology. **Zhigang Li:** Writing – review & editing, Resources, Conceptualization. **Qinhe Zhu:** Investigation, Data curation. **Changwei Liu:** Validation, Supervision, Methodology. **Haixiang He:** Validation, Investigation. **Zhixu Zhang:** Investigation. **Chuyi Zhou:** Software, Formal analysis, Data curation. **Guanying Liu:** Visualization, Investigation. **Yuqiao Zhou:** Resources, Investigation. **Senwen Deng:** Writing – review & editing, Supervision, Project administration, Funding acquisition, Conceptualization. **Shiyin Guo:** Writing – review & editing, Supervision, Project administration. **Yongzhong Chen:** Writing – review & editing, Resources, Conceptualization.

## Institutional review board statement

Animal experiments were approved by the Institutional Animal Protection and Use Committee of Hunan Agricultural University (Ethics Certificate number, 2021–2138).

## Declaration of competing interest

The authors declare that they have no known competing financial interests or personal relationships that could have appeared to influence the work reported in this paper.

## Data Availability

Data will be made available on request.

## Appendix A Supplementary data

Supplementary material Image 1
