# Supplementary material for: Extract of camellia seed cake ameliorates glycolipid metabolism disorder in mice through inhibiting ACOX1 activity
Source: Food Chem X. 2025 Jun 30;29:102707. doi: 10.1016/j.fochx.2025.102707 (PMC12275135; doi:10.1016/j.fochx.2025.102707)
Supplement: Supplementary file 1 — Supplementary material [file mmc1.docx]

**Appendix A. Supplementary data**

**Extract of camellia seed cake ameliorates glycolipid metabolism disorder in mice through inhibiting ACOX1 activity**

Bolin Chen ^a,b,e,1^, Li Ma ^b,e,1^ , Xinzhi Chen ^a,1^, Zhigang Li ^b^, Qinhe Zhu ^a^, Changwei Liu ^a^, Haixiang He ^c^, Zhixu Zhang ^d^, Chuyi Zhou ^a^ , Guanying Liu ^a^, Yuqiao Zhou ^a^, Senwen Deng ^a,e^*，Shiyin Guo^a,^* and Yongzhong Chen ^b,e^*

^a^ Hunan Engineering Research Center of Lotus Deep Processing and Nutritional Health Sciences, School of Life and Health Sciences, Hunan University of Science and Technology, Xiangtan 411201, China.

^b^ National Engineering Research Center of Oiltea Camellia, State Key Laboratory of Utilization of Woody Oil Resources, Hunan Academy of Forestry, Shao Shan South Road, No. 658, Changsha 410004, China.

^c^ Hunan Xianglian Engineering Technology Research Center, Xiangtan 411201, China.

^d^ College of Horticulture, Hunan Agricultural University, Changsha 410128, China.

^e^ Yuelushan Laboratory of Hunan Province, Changsha 410004, China.

^1^ represent these authors contributed equally to this work

**Corresponding author:**

Senwen Deng ^1,^*，Shiyin Guo^1,^* and Yongzhong Chen ^2,^*

E-mail:dswwzls@hnust.edu.cn (S.D.);

gsy@hunau.edu.cn(S.G.);

chenyongzhong@hnlky.cn (Y.C.)

Table S1 Three-factor three-level Box-Behnken experimental design for the extraction process of ACOX1 inhibitor.

Table S2 Primer sequences.

Table S3 Response surface experimental design and results.

Table S4 Response surface variance analysis results.

Table S5 Result of UHPLC-QE-Orbitrap-MS.

Table S6 Molecular docking results of compounds with ACOX1.

Table S7 The correlation analysis of liver oxidation and antioxidant indicators.

Fig. S1 Determination of the optimal extract pH (A) and extract concentration (B) for resin adsorption.

Fig. S2 IC_50_ values of camellia seed cake extract before and after purification.

Fig. S3 Total chromatogram of camellia seed cake extract in positive ion mode.

**
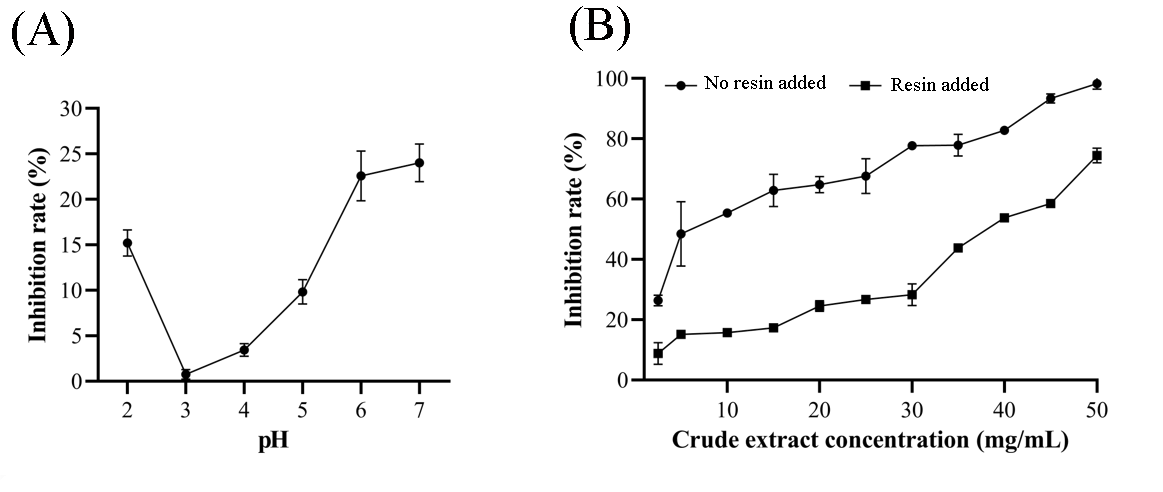
**

Fig. S1. Determination of the optimal extract pH (A) and extract concentration (B) for resin adsorption.


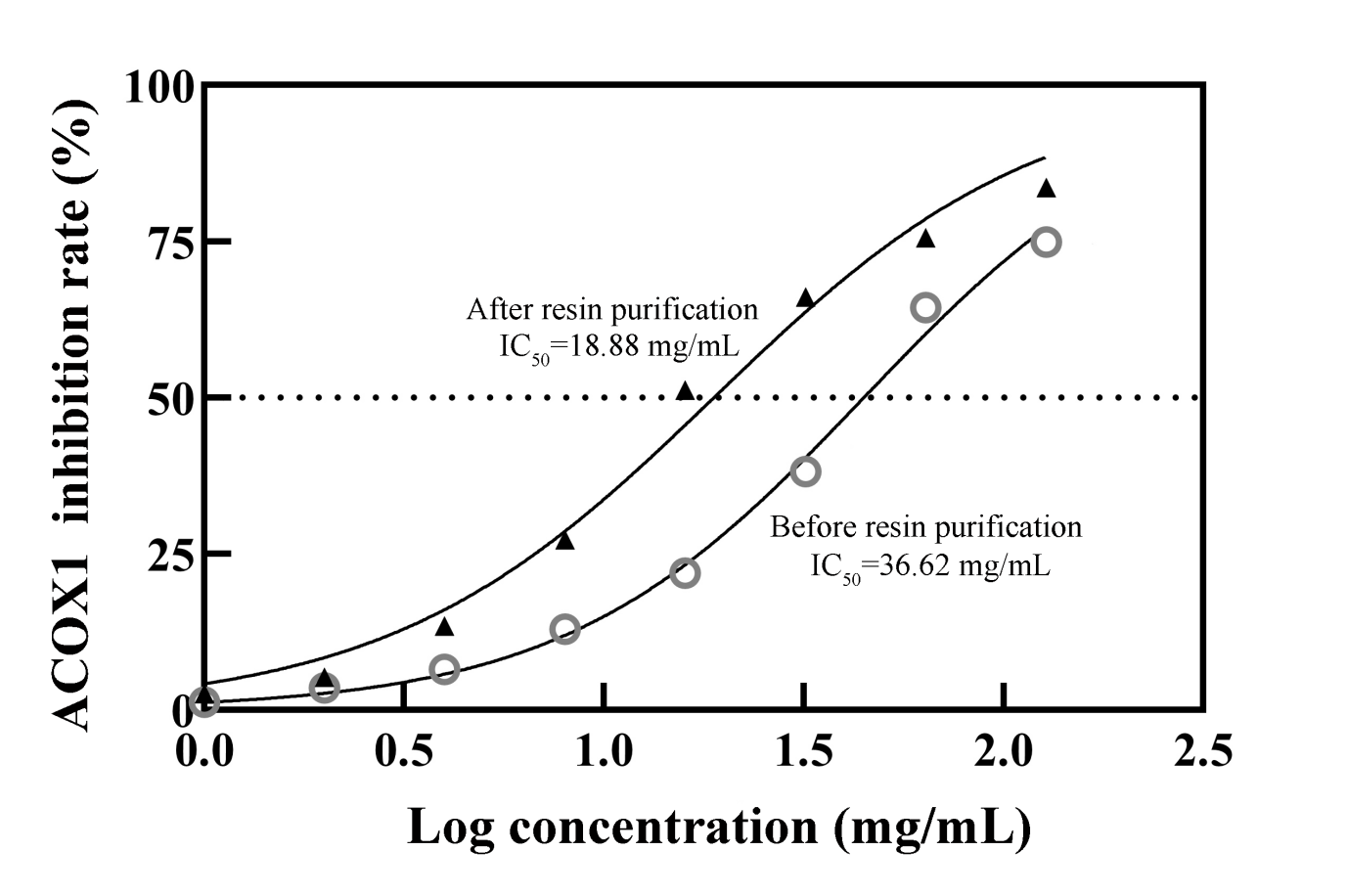


Fig. S2. IC_50_ values of camellia seed cake extract before and after purification.


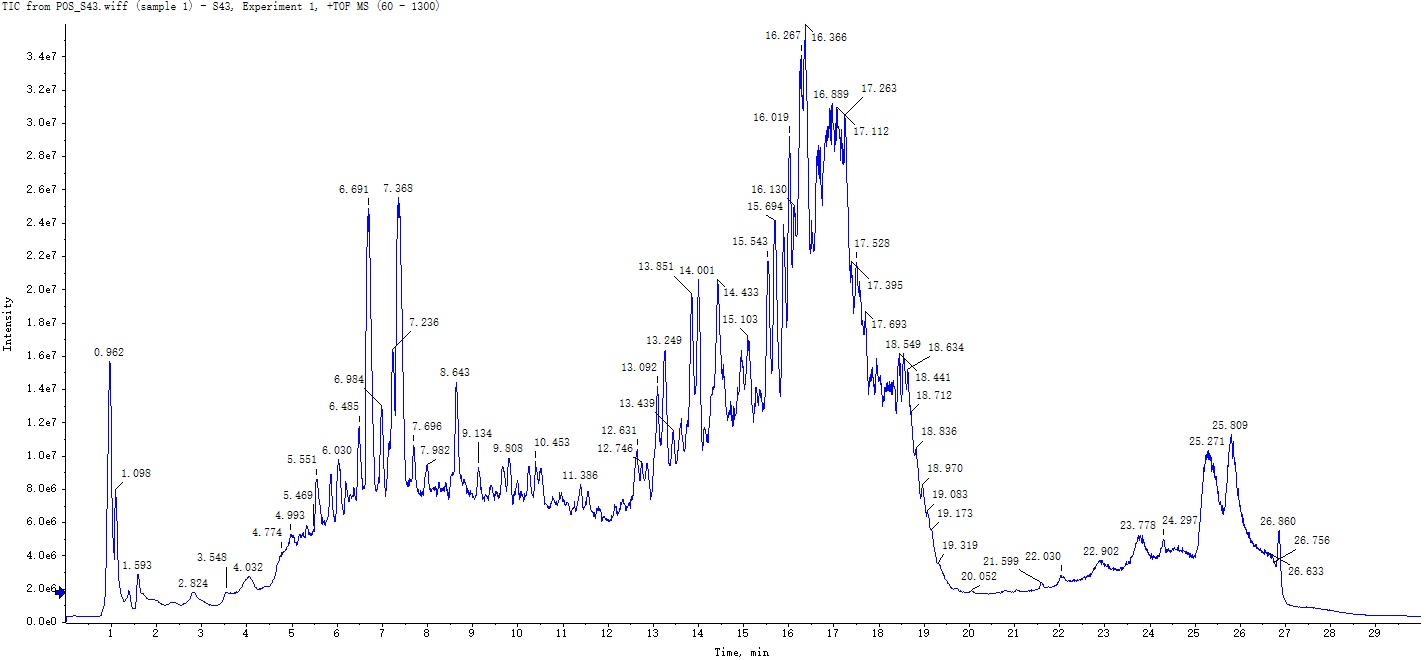


Fig. S3. Total chromatogram of camellia seed cake extract in positive ion mode.

Table S1 Three-factor three-level Box-Behnken experimental design for the extraction process of ACOX1 inhibitor.

| Levels | Factors | | |
| --- | --- | --- | --- |
|  | A Ethanol concentration/% | B extraction temperature/°C | C extraction time/min |
| -1 | 50 | 60 | 50 |
| 0 | 60 | 70 | 60 |
| 1 | 70 | 80 | 70 |

Table S2 Primer sequences.

| Genes | Forward primer | Reverse primer |
| --- | --- | --- |
| *ucp2* | 5’-CCGGTTACAGATCCAAGGAGAA-3’ | 5’-TCAGAATGGTGCCCATCACA-3’ |
| *acox1* | 5’-CTTGGATGGTAGTCCGGAGA-3’ | 5’-TGGCTTCGAGTGAGGAAGTT-3’ |
| *sirt 1* | 5’-GCTCGCCTTGCTGTGGACTTC-3’ | 5’-GTGACACAGAGATGGCTGGAACTG-3’ |
| *ubiquitin* | 5’-GCCCAGTGTTACCACCAAGAAG-3’ | 5’-GCTCTTTTTAGATACTGTGGTGAGGAA-3’ |

Table S3 Response surface experimental design and results.

| Test number | A Ethanol concentration/% | B Extraction temperature/°C | C Extraction time/min | Inhibition rate /% |
| --- | --- | --- | --- | --- |
| 1 | 1 | 1 | 0 | 80.25 |
| 2 | 0 | 0 | 0 | 83.44 |
| 3 | 0 | 0 | 0 | 83.76 |
| 4 | 1 | 0 | 1 | 72.61 |
| 5 | 0 | 1 | 1 | 81.53 |
| 6 | 0 | 0 | 0 | 84.62 |
| 7 | 0 | 1 | -1 | 68.47 |
| 8 | 1 | -1 | 0 | 74.84 |
| 9 | -1 | -1 | 0 | 81.21 |
| 10 | -1 | 0 | -1 | 73.89 |
| 11 | 0 | 0 | 0 | 83.12 |
| 12 | 0 | -1 | 1 | 76.43 |
| 13 | -1 | 1 | 0 | 77.39 |
| 14 | 0 | 0 | 0 | 84.39 |
| 15 | 1 | 0 | -1 | 77.71 |
| 16 | 0 | -1 | -1 | 77.07 |
| 17 | -1 | 0 | 1 | 78.34 |

Table S4 Response surface variance analysis results.

| Source of variance | Square sum | Degrees of freedom | Mean square | F values | P values | Significance |
| --- | --- | --- | --- | --- | --- | --- |
| Model | 336.52 | 9 | 37.39 | 8 | <0.01 | ** |
| A Ethanol concentration | 3.67 | 1 | 3.67 | 0.79 | 0.4049 |  |
| B Extraction temperature | 0.46 | 1 | 0.46 | 0.098 | 0.7639 |  |
| C Extraction time | 17.32 | 1 | 17.32 | 3.7 | 0.0957 |  |
| AB | 21.3 | 1 | 21.3 | 4.56 | 0.0702 |  |
| AC | 22.8 | 1 | 22.8 | 4.88 | 0.0629 |  |
| BC | 46.92 | 1 | 46.92 | 10.04 | <0.05 | * |
| A^2^ | 38.92 | 1 | 38.92 | 8.33 | <0.05 | * |
| B^2^ | 33.08 | 1 | 33.08 | 7.08 | <0.05 | * |
| C^2^ | 131.48 | 1 | 131.48 | 28.13 | <0.01 | ** |
| Residual | 32.72 | 7 | 4.67 |  |  |  |
| Lack of fit | 24.91 | 3 | 8.3 | 4.25 | 0.0979 |  |

* *P* < 0.05；** *P* < 0.01; R^2^=0.9114；R^2^_Adj_ =0.7975；CV=2.74%.

Table S5 Result of UHPLC-QE-Orbitrap-MS.

| Number | Retention time/min | Compound | Formula | Mass-to-Charge Ratio | Adducts | Height | Area | Gaussian | Signal-to-Noise Ratio |
| --- | --- | --- | --- | --- | --- | --- | --- | --- | --- |
| 1 | 7.3737 | Kaempferol | C_15_H_10_O_6_ | 287.0567 | [M+H]+ | 4863430 | 50058200 | 0.9401399 | 35661.62 |
| 2 | 7.3636 | Kaempferol-3-*O*-rutinoside | C_27_H_30_O_15_ | 595.1697 | [M+H]+ | 2969629 | 21224270 | 0.8324648 | 9510.732 |
| 3 | 7.3636 | Luteolin-4'-*O*-glucoside | C_21_H_20_O_11_ | 449.1087 | [M+H]+ | 1387052 | 13649440 | 0.8313316 | 6406.977 |
| 4 | 7.7027 | Apigenin-8-C-glucoside-2'-rhamnoside | C_27_H_30_O_14_ | 579.1746 | [M+H]+ | 1969629 | 13649440 | 0.9151322 | 3984.952 |
| 5 | 6.6981 | Cyanidin-3-*O*-glucoside | C_21_H_21_ClO_11_ | 449.109 | [M]+ | 1504246 | 10920560 | 0.7669032 | 4640.516 |
| 6 | 7.2392 | Kaempferol-3-*O*-galactoside-7-O-rhamnoside | C_15_H_10_O_6_ | 595.1698 | [M+H]+ | 1689237 | 8214256 | 0.8584255 | 8614.185 |
| 7 | 8.6423 | Luteolin-7-*O*-glucoside | C_21_H_20_O_11_ | 449.1093 | [M+H]+ | 1325315 | 8162627 | 0.8091771 | 4010.488 |
| 8 | 6.9843 | Eleutheroside E | C_34_H_46_O_18_ | 765.2612 | [M+Na]+ | 803283.5 | 4533863 | 0.744468 | 8394.221 |
| 9 | 25.8076 | Phthalic acid | C_8_H_6_O_4_ | 167.0336 | [M+H]+ | 142528.8 | 3213490 | 0.6266257 | 615.7243 |
| 10 | 7.7027 | Isovitexin | C_21_H_20_O_10_ | 433.1155 | [M+H]+ | 512857.6 | 2522281 | 0.8312324 | 2243.618 |
| 11 | 4.6738 | Methyl nicotinic acid | C_7_H_7_NO_2_ | 138.0553 | [M+H]+ | 389540.6 | 2413820 | 0.8607289 | 3350.61 |
| 12 | 8.2607 | Cyanidin 3-(2G-glucosylrutinoside) | C_33_H_41_ClO_20_ | 757.2208 | [M]+ | 426929.3 | 2047834 | 0.8371401 | 3199.756 |
| 13 | 21.5981 | Tri(butoxyethyl)phosphate | C_18_H_39_O_7_P | 399.2522 | [M+H]+ | 278101.9 | 2014508 | 0.74984 | 216.0478 |
| 14 | 8.5241 | 5,7-dihydroxy-2-(4-hydroxyphenyl)-4H-chromen-4-one | C_15_H_10_O_5_ | 271.0618 | [M+H]+ | 375904.2 | 1784485 | 0.856119 | 2663.197 |
| 15 | 8.7486 | 3,4-Dimethoxycinnamic acid | C_11_H_12_O_4_ | 191.0705 | [M+H-H2O]+ | 329927.9 | 1684850 | 0.8372128 | 1796.823 |
| 16 | 6.6573 | Camelliaside A | C_33_H_40_O_20_ | 779.2033 | [M+Na]+ | 377934.6 | 1609719 | 0.772747 | 2419.388 |
| 17 | 1.0222 | Methylophiopogonanone A | C_19_H_18_O_6_ | 365.1055 | [M+Na]+ | 377528.3 | 1563404 | 0.9445215 | 1751.074 |
| 18 | 22.0225 | angoletin | C_18_H_20_O_4_ | 301.1416 | [M+H]+ | 133883.4 | 1549765 | 0.6847394 | 249.2245 |
| 19 | 9.1341 | Episyringaresinol 4'-*O*-beta-D-glncopyranoside | C_28_H_36_O_13_ | 603.2045 | [M+Na]+ | 267613.8 | 1320655 | 0.8867141 | 1757.173 |
| 20 | 9.1448 | Apigenin-7-*O*-neohesperidoside | C_27_H_30_O_14_ | 579.1714 | [M+H]+ | 247101.1 | 1205029 | 0.8709173 | 496.0887 |
| 21 | 7.7997 | Vitexin | C_21_H_20_O_10_ | 433.1155 | [M+H]+ | 203145.1 | 1050570 | 0.8916296 | 417.271 |
| 22 | 7.3227 | Camelliaside B | C_32_H_38_O_19_ | 749.1915 | [M+Na]+ | 263475.4 | 1036822 | 0.9242058 | 1626.457 |
| 23 | 7.9437 | nicotiflorin | C_27_H_30_O_15_ | 595.1669 | [M+H]+ | 163386.6 | 854376.7 | 0.7935749 | 673.6829 |
| 24 | 1.0644 | 4-Guanidinobutyric acid | C_5_H_11_N_3_O_2_ | 146.0917 | [M+H]+ | 179178.4 | 703062.8 | 0.9491023 | 1786.976 |
| 25 | 6.6879 | afzelin | C_21_H_20_O_10_ | 433.1125 | [M+H]+ | 73147.06 | 639111.9 | 0.8722058 | 524.6758 |
| 26 | 16.4615 | Hederacoside D | C_53_H_86_O_22_ | 1097.554 | [M+Na]+ | 99363.94 | 483192.1 | 0.8222349 | 1936.359 |
| 27 | 7.7027 | clitorin | C_33_H_40_O_19_ | 763.208 | [M+Na]+ | 90055.25 | 459105.6 | 0.9665988 | 918.593 |
| 28 | 9.3537 | Puerarin | C_21_H_20_O_9_ | 417.1195 | [M+H]+ | 76468.56 | 439467 | 0.742152 | 366.9667 |
| 29 | 1.0117 | betaine | C_5_H_11_NO_2_ | 140.069 | [M+Na]+ | 1.04E+05 | 408165.6 | 0.9304209 | 1167.081 |
| 30 | 7.6485 | Isorhamnetin | C_16_H_12_O_7_ | 317.0676 | [M+H]+ | 74801.06 | 362926.1 | 0.8887818 | 377.3801 |
| 31 | 13.7146 | Vinpocetine | C_22_H_26_N_2_O_2_ | 373.1871 | [M+Na]+ | 60212.94 | 338184.4 | 0.9014841 | 202.9812 |
| 32 | 8.5241 | Pelargonidin-3-*O*-glucoside | C_21_H_21_O_10_ | 433.1127 | [M]+ | 61476.13 | 291371.3 | 0.9242389 | 438.8663 |
| 33 | 6.0758 | quercetin-3-*O*-glc-1-3-rham-1-6-glucoside | C_27_H_30_O_17_ | 773.2173 | [M+H]+ | 57836.19 | 281920.9 | 0.8383825 | 437.2853 |
| 34 | 22.0980 | lithocholic acid | C_24_H_40_O_3_ | 359.2949 | [M-H2O+H]+ | 40684.19 | 259044.5 | 0.875771 | 268.3981 |
| 35 | 9.7819 | 2''-Rhamnosylvitexin | C_27_H_30_O_14_ | 601.1545 | [M+Na]+ | 48769.56 | 253771.5 | 0.9052684 | 247.9664 |
| 36 | 17.5584 | Platycodin D | C_57_H_92_O_28_ | 1225.597 | [M+H]+ | 46772.56 | 246641.2 | 0.7671066 | 704.0203 |
| 37 | 7.4449 | Isovitexin 2''-*O*-arabinoside | C_26_H_28_O_14_ | 565.1555 | [M+H]+ | 25105.5 | 215654.9 | 0.7731953 | 185.9301 |
| 38 | 7.7027 | Kaempferitrin | C_27_H_30_O_14_ | 601.154 | [M+Na]+ | 36398.56 | 209006.6 | 0.9396304 | 183.814 |
| 39 | 1.3584 | Adenosine | C_10_H_13_N_5_O_4_ | 268.1056 | [M+H]+ | 45239.31 | 205072.6 | 0.9240324 | 393.0523 |
| 40 | 8.2497 | Panasenoside | C_27_H_30_O_16_ | 611.1611 | [M+H]+ | 32626.44 | 174254.9 | 0.8862674 | 45.37971 |
| 41 | 9.4531 | Mitoridine | C_20_H_22_N_2_O_2_ | 345.1513 | [M+Na]+ | 31381.94 | 170887.8 | 0.8798833 | 149.009 |
| 42 | 6.3683 | Pinoresinol diglucoside | C_32_H_42_O_16_ | 705.2354 | [M+Na]+ | 31988.88 | 170849.6 | 0.9731492 | 222.5987 |
| 43 | 8.9133 | Isorhamnetin | C_16_H_12_O_7_ | 317.0674 | [M+H]+ | 32247.25 | 170725.3 | 0.9151397 | 77.22941 |
| 44 | 6.0758 | Quercetin | C_15_H_10_O_7_ | 303.0485 | [M+H]+ | 33907.19 | 170585.1 | 0.8136455 | 161.7241 |
| 45 | 8.3373 | coniferin | C_16_H_22_O_8_ | 365.1219 | [M+Na]+ | 30388.25 | 168575.8 | 0.9565392 | 139.113 |
| 46 | 9.0681 | Quercitrin | C_21_H_20_O_11_ | 471.0903 | [M+Na]+ | 33207.94 | 162522.4 | 0.8753098 | 175.9294 |
| 47 | 1.9240 | 4-Methyl-5-thiazoleethanol | C_6_H_9_NOS | 144.0484 | [M+H]+ | 32045.69 | 153596 | 0.8498837 | 242.9743 |
| 48 | 7.3737 | Cyanidin-3-*O*-alpha-arabinopyranoside | C_20_H_19_O_10_ | 419.0949 | [M]+ | 14317.44 | 134765.8 | 0.7770134 | 108.9222 |
| 49 | 5.8937 | Kaempferol 3-*O*-sophoroside | C_27_H_30_O_16_ | 611.1652 | [M+H]+ | 22079.75 | 129942.2 | 0.9617518 | 30.55303 |
| 50 | 2.0743 | Ecgonine | C_9_H_15_NO_3_ | 186.1121 | [M+H]+ | 22965.25 | 126392 | 0.8837208 | 137.303 |
| 51 | 9.1772 | Phloretin | C_15_H_14_O_5_ | 275.0912 | [M+H]+ | 24154.25 | 126382.6 | 0.9333926 | 73.87251 |
| 52 | 16.6938 | Ciwujianoside B | C_59_H_94_O_24_ | 1211.578 | [M+Na]+ | 18851.56 | 119365.9 | 0.8587058 | 504.4702 |
| 53 | 7.6375 | Licochalcone A | C_21_H_22_O_4_ | 361.1396 | [M+Na]+ | 19322.94 | 119281 | 0.8315328 | 128.8104 |
| 54 | 5.8072 | Cimifugin 4'-O-beta-D-glucopyranoside | C_22_H_28_O_11_ | 469.1696 | [M+H]+ | 23568.25 | 118141.1 | 0.8382698 | 154.4733 |
| 55 | 7.6049 | Nicotinic acid | C_6_H_5_NO_2_ | 124.0396 | [M+H]+ | 14312 | 117400.79 | 0.8644354 | 66.23125 |
| 56 | 1.0326 | Trigonelline | C_7_H_7_NO_2_ | 138.055 | [M+H]+ | 23623.13 | 114735.4 | 0.7538766 | 202.892 |
| 57 | 7.6594 | Isorhamnetin-3*O*-glucoside | C_22_H_20_O_11_ | 479.1211 | [M+H]+ | 19629.94 | 113833.09 | 0.6829548 | 79.01806 |
| 58 | 9.7819 | Apigenin 7-*O*-glucoside | C_21_H_20_O_10_ | 433.113 | [M+H]+ | 16329.19 | 113335.3 | 0.8837898 | 110.2489 |
| 59 | 6.6040 | hyperoside | C_21_H_20_O_10_ | 465.1049 | [M+H]+ | 22534.56 | 107765.1 | 0.7751194 | 119.695 |
| 60 | 10.3257 | 3 5 7-trihydroxy-4'-methoxyflavone | C_16_H_12_O_6_ | 301.0707 | [M+H]+ | 19928.75 | 107744.7 | 0.8357984 | 38.0626 |
| 61 | 16.3048 | Polyphyllin VII | C_45_H_72_O_16_ | 1053.533 | [M+Na]+ | 17420.56 | 104645.3 | 0.801152 | 337.6746 |
| 62 | 8.9133 | Isorhamnetin-3-*O*-rutinoside | C_27_H_30_O_14_ | 647.162 | [M+Na]+ | 17185.19 | 100728.4 | 0.9396344 | 123.6761 |
| 63 | 17.3051 | Dipsacoside B | C_43_H_68_O_19_ | 1097.543 | [M+Na]+ | 18185.31 | 98726.12 | 0.8898845 | 299.8259 |
| 64 | 14.5722 | Protodioscin | C_45_H_72_O_16_ | 1071.539 | [M+Na]+ | 13791.75 | 96026.2 | 0.7858644 | 240.0483 |
| 65 | 17.7484 | Methyl protodioscin | C_45_H_72_O_16_ | 1085.557 | [M+Na]+ | 15274.5 | 90865.89 | 0.8960122 | 231.7205 |
| 66 | 8.5241 | 3-Feruloyl-1-Sinapoyl sucrose | C_55_H_84_O_26_ | 747.2136 | [M+Na]+ | 18114.31 | 88939.49 | 0.8626378 | 150.0334 |
| 67 | 9.8360 | Mirificin | C_55_H_84_O_26_ | 549.1604 | [M+H]+ | 14300.25 | 85410.13 | 0.9417468 | 64.28427 |
| 68 | 9.6512 | Peonidin-3-*O*-glucoside | C_27_H_30_O_16_ | 463.1265 | [M]+ | 9685.625 | 82859.38 | 0.7667052 | 46.8861 |
| 69 | 14.6356 | 14-hydroxysprengerinin C | C_27_H_30_O_16_ | 893.4559 | [M+Na]+ | 12430.94 | 82399.52 | 0.9180376 | 109.3245 |
| 70 | 7.7450 | Rutin | C_27_H_30_O_16_ | 633.137 | [M+Na]+ | 13495 | 81385.71 | 0.7831781 | 64.09145 |
| 71 | 13.8629 | Emetine Hydrochloride | C_34_H_43_NO_9_·HCl | 517.2814 | [M+H]+ | 11009.06 | 81239.4 | 0.8773491 | 29.85734 |
| 72 | 13.8839 | Glycyrrhizic acid | C_42_H_62_O_16_ | 845.3954 | [M+Na]+ | 11840.44 | 81118.6 | 0.8322121 | 153.1382 |
| 73 | 7.5179 | Isorhamnetin | C_16_H_12_O_7_ | 317.0677 | [M+H]+ | 16837.81 | 79704.59 | 0.9308954 | 86.07909 |
| 74 | 19.9129 | Chenodeoxycholic acid | C_24_H_40_O_4_ | 357.2792 | [M+H-2H2O]+ | 12182 | 76312.36 | 0.7751607 | 92.01705 |
| 75 | 10.3148 | Robinin | C_27_H_30_O_16_ | 763.2112 | [M+Na]+ | 16039.06 | 76213.09 | 0.8388799 | 93.97515 |
| 76 | 18.4998 | Tris(1-chloro-2-propyl)phosphate | C_6_H_14_Cl_3_O_4_P | 327.0081 | [M+H]+ | 6299.125 | 75447.07 | 0.6351532 | 52.85184 |
| 77 | 9.3537 | Datiscetin-3-*O*-rutinoside | C_27_H_30_O_16_ | 595.1641 | [M+H]+ | 7766.75 | 75113.65 | 0.7230909 | 23.89288 |
| 78 | 9.5964 | Neodiosmin | C_27_H_32_O_15_ | 609.178 | [M+H]+ | 10578.06 | 72919.39 | 0.8699352 | 63.46999 |
| 79 | 4.6183 | Forsythoside E | C_27_H_30_O_15_ | 485.1661 | [M+Na]+ | 9954.188 | 65342.17 | 0.861789 | 48.46118 |
| 80 | 8.9574 | aflatoxin B1 | C_17_H_12_O_6_ | 313.0715 | [M+H]+ | 12019.13 | 64894.15 | 0.8039533 | 57.04245 |
| 81 | 8.1065 | Grosvenorine | C_38_H_41_NO_10_ | 763.2039 | [M+Na]+ | 11817.38 | 64883.75 | 0.9569339 | 65.1158 |
| 82 | 9.3869 | Dihydrokaempferol | C_15_H_12_O_6_ | 289.0715 | [M+H]+ | 10687.13 | 64384.19 | 0.7804584 | 29.26816 |
| 83 | 9.3869 | Eriocitrin | C_21_H_20_O_10_ | 619.1636 | [M+Na]+ | 11144.31 | 63573.35 | 0.8978397 | 32.94195 |
| 84 | 5.4932 | Kaempferol-3-*O*-alpha-L-rhamnoside | C_21_H_20_O_10_ | 433.1127 | [M+H]+ | 11565.75 | 63472.1 | 0.9227036 | 79.83485 |
| 85 | 9.2097 | Brusatol | C_39_H_54_O_16_ | 543.1819 | [M+Na]+ | 8257.125 | 61867.04 | 0.8170045 | 19.90526 |
| 86 | 20.6382 | Drofenine | C_20_H_18_O_9_ | 318.241 | [M+H]+ | 11144.13 | 61403.58 | 0.9631758 | 64.78339 |
| 87 | 13.1316 | Esculentoside H | C_55_H_84_O_26_ | 1011.477 | [M+Na]+ | 7519.25 | 59434.23 | 0.8785951 | 122.3366 |
| 88 | 14.9325 | Indole-3-carbinol | C_8_H_9_NO | 130.065 | [M+H-H2O]+ | 6486.188 | 57440.96 | 0.8691838 | 54.52475 |
| 89 | 11.0133 | Esculentoside A | C_55_H_84_O_26_ | 849.423 | [M+Na]+ | 8961.813 | 55917.53 | 0.8514301 | 116.0589 |
| 90 | 9.8467 | harpagoside | C_60_H_92_O_28_ | 495.1819 | [M+H]+ | 9258.625 | 55285.41 | 0.8837547 | 18.82979 |
| 91 | 11.7180 | Angelol A | C_27_H_30_O_16_ | 399.1404 | [M+Na]+ | 9551.438 | 55160.54 | 0.9433336 | 6.984042 |
| 92 | 9.1231 | Naringenin | C_15_H_12_O_6_ | 273.0771 | [M+H]+ | 10086.19 | 54543.15 | 0.8952867 | 39.39444 |
| 93 | 16.6623 | Kudinoside D | C_57_H_90_O_26_ | 931.4673 | [M+Na]+ | 8840.875 | 52725.81 | 0.8342956 | 48.44019 |
| 94 | 15.8950 | Desapioplatycodin D | C_30_H_42_O_14_ | 1115.521 | [M+Na]+ | 6749.063 | 52532.34 | 0.8645285 | 120.1676 |
| 95 | 9.4091 | Rhoifolin | C_27_H_32_O_16_ | 601.1548 | [M+Na]+ | 8821.938 | 51589.83 | 0.8439482 | 41.56105 |
| 96 | 10.4565 | Agarotetrol | C_15_H_20_O_7_ | 341.1032 | [M+Na]+ | 6403.5 | 51391.14 | 0.8592558 | 22.82199 |
| 97 | 8.1175 | Delphinidin-3-*O*-beta-glucopyranoside | C_21_H_19_O_10_ | 465.1015 | [M]+ | 7900.875 | 50396.24 | 0.949226 | 39.09632 |
| 98 | 7.8550 | Ellagic acid | C_14_H_6_O_8_ | 303.0161 | [M+H]+ | 8184.563 | 49567.45 | 0.8513823 | 89.85358 |
| 99 | 8.9133 | Pinoresinol 4-*O*-glucoside | C_20_H_20_O_9_ | 543.1887 | [M+Na]+ | 8293.563 | 49069.73 | 0.890372 | 20.44407 |
| 100 | 8.9353 | Etoposide | C_29_H_32_O_13_ | 589.1888 | [M+H]+ | 8646.813 | 48971.86 | 0.7548742 | 50.3005 |
| 101 | 8.9683 | Toosendanin | C_55_H_84_O_26_ | 597.2313 | [M+Na]+ | 5776.5 | 47844.16 | 0.8016252 | 10.02358 |
| 102 | 12.7035 | Polygalasaponin F | C_55_H_84_O_26_ | 1113.542 | [M+Na]+ | 4393.313 | 43401.46 | 0.7490438 | 85.42867 |
| 103 | 9.3869 | Galangin | C_21_H_20_O_10_ | 271.0597 | [M+H]+ | 6690.5 | 43181.06 | 0.7074044 | 41.0284 |
| 104 | 6.0652 | Delphinidin 3-galactoside | C_21_H_19_O_10_ | 465.1021 | [M]+ | 7565.063 | 42678.64 | 0.9143782 | 38.83332 |
| 105 | 6.8873 | Syringaresnol-4-*O*-beta-D-apiofuranosy | C_21_H_20_O_9_ | 735.2487 | [M+Na]+ | 8241.188 | 42236.36 | 0.9522682 | 54.85205 |
| 106 | 11.2531 | Kaempferol-3-*O*-glucoside | C_21_H_20_O_10_ | 449.109 | [M+H]+ | 4783.875 | 41404.75 | 0.8493776 | 15.74777 |
| 107 | 9.1448 | Apigenin | C_15_H_10_O_4_ | 271.0595 | [M+H]+ | 7026.563 | 40228.93 | 0.9553576 | 43.2155 |
| 108 | 6.3138 | Glucosylgentiopicroside | C_16_H_22_O_9_ | 541.1492 | [M+Na]+ | 7128.5 | 39693.89 | 0.53082 | 29.25875 |
| 109 | 11.7617 | Betulin | C_27_H_44_O_6_ | 443.3884 | [M+H]+ | 7321.063 | 39663.48 | 0.6819876 | 27.04965 |
| 110 | 7.6267 | aloesin | C_45_H_70_O_16_ | 395.1338 | [M+H]+ | 4835.438 | 39024.94 | 0.7953878 | 20.79412 |
| 111 | 5.7300 | Kaempferol-3-*O*-robinoside-7-*O*-rhamnoside | C_22_H_20_O_10_ | 741.2252 | [M+H]+ | 3978.625 | 38758.86 | 0.9295667 | 29.5181 |
| 112 | 13.6292 | Tomatine | C_45_H_69_O_18_ | 1034.563 | [M+H]+ | 6506.938 | 38000.6 | 0.8882456 | 124.3801 |
| 113 | 6.3575 | Cyanidin-3-*O*-(2''-*O*-beta-xylopyranosyl-beta-glucopyranoside)-5-*O*-beta-glucopyranoside | C_33_H_41_O_20_ | 743.2 | [M]+ | 5332.188 | 37995.5 | 0.893339 | 39.88295 |
| 114 | 9.9012 | Isoshaftoside | C_21_H_20_O_10_ | 587.1425 | [M+Na]+ | 6086.625 | 37851.15 | 0.5878361 | 27.20802 |
| 115 | 2.1907 | Phenylalanine | C_9_H_11_NO_2_ | 166.0859 | [M+H]+ | 3875.688 | 36906.59 | 0.801762 | 17.44075 |
| 116 | 5.4932 | Kaempferol-3-*O*-beta-glucopyranosyl-7-*O*-alpha-rhamnopyranoside | C_21_H_20_O_10_ | 595.1669 | [M+H]+ | 4412.25 | 36709.97 | 0.842702 | 13.01401 |
| 117 | 7.6704 | Dehydrocorydalin | C_20_H_19_NO_4_ | 367.1723 | [M+H]+ | 4668.688 | 34998.98 | 0.7499459 | 22.08056 |
| 118 | 1.7290 | 1-Methyladenosine | C_11_H_13_N_5_O_4_ | 282.1194 | [M+H]+ | 6687.938 | 34979.46 | 0.7934576 | 41.43936 |
| 119 | 10.0755 | Kaempferol-3-*O*-glucoside-3''-rhamnoside | C_21_H_20_O_10_ | 595.1696 | [M+H]+ | 4029.813 | 34383.69 | 0.8166687 | 11.09048 |
| 120 | 17.4529 | Jujuboside B | C_55_H_84_O_26_ | 1067.537 | [M+Na]+ | 4413.688 | 33138.63 | 0.821805 | 70.94336 |
| 121 | 4.7854 | Cyanidin-3,5-di-*O*-glucoside | C_33_H_41_O_20_ | 611.1651 | [M]+ | 4135.438 | 32885.66 | 0.754813 | 5.195618 |
| 122 | 11.8922 | Jujuboside D | C_55_H_84_O_26_ | 1229.591 | [M+Na]+ | 3636.875 | 32258.78 | 0.9356373 | 78.6663 |
| 123 | 6.7184 | Indolelactic acid | C_10_H_9_NO_2_ | 206.0802 | [M+H]+ | 3381.563 | 30300.91 | 0.8553936 | 14.92763 |
| 124 | 19.1473 | Lauryl diethanolamide | C_20_H_39_NO_2_ | 288.2546 | [M+H]+ | 3573.313 | 26833.76 | 0.8046237 | 30.56874 |
| 125 | 5.1624 | 6,7-DIHYDROXYCOUMARIN | C_9_H_6_O_3_ | 179.0314 | [M+H]+ | 4205.375 | 26637.47 | 0.9654118 | 18.75555 |
| 126 | 8.4910 | Hispiduloside | C_27_H_30_O_16_ | 485.1102 | [M+Na]+ | 4110.563 | 25274.64 | 0.8894736 | 19.37617 |
| 127 | 5.6970 | Cyanidin-3-*O*-rhamnoside | C_21_H_19_O_10_ | 433.113 | [M]+ | 3260.313 | 24780.42 | 0.7081177 | 15.1605 |
| 128 | 2.9483 | Loganin | C_27_H_32_O_16_ | 413.142 | [M+Na]+ | 1639.688 | 24373.37 | 0.6929008 | 7.557736 |
| 129 | 9.0569 | Apigenin-7-*O*-(2G-rhamnosyl)gentiobioside | C_27_H_30_O_16_ | 763.2029 | [M+Na]+ | 4059.063 | 24098.13 | 0.8098906 | 20.13415 |
| 130 | 4.0535 | 7-*O*-Methyl morroniside | C_22_H_26_O_9_ | 443.152 | [M+Na]+ | 3454.75 | 23061.91 | 0.9161881 | 19.24133 |
| 131 | 1.4020 | L-Pyroglutamic acid | C_5_H_7_NO_2_ | 130.0506 | [M+H]+ | 3519.625 | 17487.12 | 0.713697 | 28.40718 |
| 132 | 7.7777 | resveratrol | C_14_H_12_O_3_ | 229.0855 | [M+H]+ | 3078.188 | 17089.17 | 0.8841614 | 12.76193 |
| 133 | 5.9588 | Norharman | C_10_H_9_NO | 169.0764 | [M+H]+ | 2474.5 | 16725.49 | 0.9404027 | 16.19348 |
| 134 | 16.0735 | Asiaticoside | C_35_H_52_O_16_ | 981.5032 | [M+Na]+ | 2789.563 | 15715.52 | 0.8125768 | 25.58434 |
| 135 | 5.6315 | Agnuside | C_21_H_20_O_10_ | 467.1514 | [M+H]+ | 2276.063 | 14663.01 | 0.908162 | 17.08621 |
| 136 | 7.9105 | N-acetylphenylalanine | C_9_H_11_NO_2_ | 208.0969 | [M+H]+ | 1894.875 | 13556.53 | 0.9488649 | 9.137722 |
| 137 | 6.4450 | Nepetin-7-glucoside | C_27_H_32_O_16_ | 501.1005 | [M+Na]+ | 2093.875 | 12452.01 | 0.8179831 | 7.387987 |
| 138 | 21.0101 | Decahydrogambogic Acid | C_15_H_24_O_4_ | 639.3929 | [M+H]+ | 1313.25 | 10985.35 | 0.8864097 | 3.727984 |

Table S6 Molecular docking results of compounds with ACOX1.

| Ligand Name | MM/GBSA ΔG Bind (kcal/mol) | Docking score  (kcal/mol) |
| --- | --- | --- |
| Luteolin-7-*O*-glucoside | -46.15 | -9.783 |
| Luteolin-4'-*O*-glucoside | -41.29 | -10.796 |
| Kaempferol | -38.42 | -7.337 |
| Kaempferol-3-*O*-galactoside-7-*O*-rhamnoside | -37.37 | -9.372 |
| 5,7-dihydroxy-2-(4-hydroxyphenyl)-4H-chromen-4-one | -34.59 | -7.207 |
| 3,4-Dimethoxycinnamic acid | -31.53 | -5.068 |
| Methylophiopogonanone A | -30.01 | -6.049 |
| Kaempferol-3-*O*-rutinoside | -26.44 | -10.139 |
| Eleutheroside E | -22.71 | -11.365 |
| Apigenin-8-C-glucoside-2'-rhamnoside | -20.81 | -9.618 |
| Camelliaside A | -16.81 | -10.802 |
| Tri(butoxyethyl)phosphate | -11.31 | -3.926 |
| Isovitexin | -9.31 | -2.54 |
| Episyringaresinol 4'-*O*-beta-D-glucopyranoside | -8.31 | -3.54 |
| Cyanidin-3-*O*-glucoside | -7.65 | -5.22 |
| Phthalic acid | -7.11 | -5.12 |
| Apigenin-7-*O*-neohesperidoside | -6.98 | -2.41 |
| Methyl nicotinic acid | -5.34 | -3.65 |
| Cyanidin 3-(2G-glucosylrutinoside) | -5.23 | -3.25 |
| Angoletin | -3.21 | -5.31 |

Table S7 The correlation analysis of liver oxidation and antioxidant indicators.

| Oxidation and antioxidant indicators | Liver CAT | Liver GSH-Px | Liver T-SOD | Liver H_2_O_2_ | Liver MDA |
| --- | --- | --- | --- | --- | --- |
| Liver CAT | 1 |  |  |  |  |
| Liver GSH-Px | 0.623* | 1 |  |  |  |
| Liver T-SOD | 0.627* | 0.685* | 1 |  |  |
| Liver H_2_O_2_ | -0.448 | -0.852** | -0.848** | 1 |  |
| Liver MDA | -0.462 | -0.808** | -0.628* | 0.819** | 1 |

* *P* < 0.05；** *P* < 0.01.
